# Cobalt catalysed aminocarbonylation of thiols in batch and flow for the preparation of amides†

**DOI:** 10.1039/d1ra04736a

**Published:** 2021-09-13

**Authors:** Jose Maria Orduña, Gema Domínguez, Javier Pérez-Castells

**Affiliations:** Department of Chemistry and Biochemistry, Facultad de Farmacia, Universidad San Pablo-CEU, CEU Universities, Urbanización Montepríncipe 28660 Boadilla del Monte Madrid Spain jpercas@ceu.es

## Abstract

The synthesis of amides from thiols through a cobalt-catalyzed aminocarbonylation is shown. After optimizing all the reaction parameters, the methodology makes possible the obtention of amides with variable yields, while competing reactions such as the formation of disulfides and ureas can be limited. The process works well with aromatic thiols with electron donating groups (EDG) whereas other thiols give reaction with lower yields. The previous process has been transferred and optimized into flow equipment, thus allowing using less CO in a safer way, and permitting the scaling up of the synthesis. Two drugs, moclobemide and itopride were prepared with this methodology, albeit only in the second case with good results. A mechanistic pathway is proposed.

## Abstract 2

The synthesis of amides from thiols through a cobalt-catalyzed aminocarbonylation is shown.

## Graphical abstract

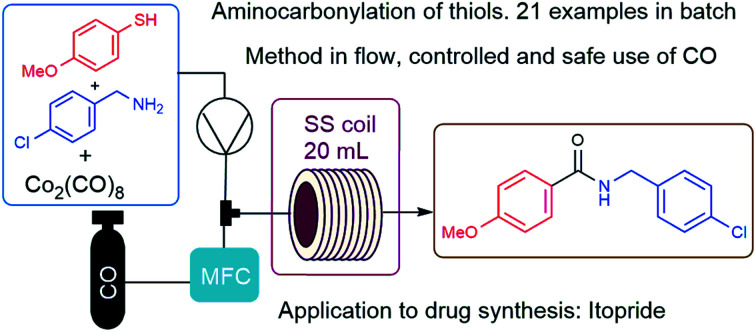

## 1 Introduction

The formation of the amide bond is of particular relevance, since, in addition to being part of the primary structure of proteins, it is a functional group that frequently appears in pharmacologically interesting compounds.^1^ Alternatives to the synthesis of amides through condensation of acids and amines include multicomponent processes such as aminocarbonylations.^2^ The palladium catalysed reaction of aryl halides or activated alkyl halides and amines, under a CO atmosphere has been extensively studied in the literature including intramolecular examples.^3^ An aminocarbonylation protocol under mild conditions has also been developed using rhodium catalysts, CO, aliphatic azides as nitrene precursors, and aryl aromatic compounds, to give rise to the corresponding amides.^4^

In 1986 Alper *et al.* described a synthesis of amides from thiols, imines and CO mediated by Co_2_(CO)_8_.^5^ The reaction conditions were harsh: high CO pressure (55 to 66 atm), and high temperature (180 °C) for 12 hours. The desired amide was isolated in moderate yields (30–59%) together with a thioester, a secondary amide and the disulfide of the starting thiol. This group developed as well alkoxycarbonylations using high CO pressures, high temperature and long reaction times.^6^ Furthermore, it was observed that these reactions were not operative under anhydrous conditions or with other catalysts such as Fe_3_(CO)_12_ and [Rh(CO)_2_]_2_.

More recently, some cobalt complex catalyzed aminocarbonylations have been published. Thus, Veatch *et al.* used visible light to promote a Co_2_(CO)_8_ aminocarbonylation of aryl halides and primary and secondary amines.^7^ Sargent *et al.* disclosed an aminocarbonylation on aliphatic tosylates, with high pressures of CO and 10 mol% of Co_2_(CO)_8_.^8^ This process was effective with both primary and secondary amines. Carbonylation of aryl halides with 2-aminopyridines using Pd–Co(CO)_4_ bimetallic catalysis has been recently disclosed.^9^

Herein, we describe a methodology for obtaining amides using thiols and amines as starting substrates through a cobalt mediated carbonylative coupling. Both batch and flow procedures will be used. The latter allow the application of harsh conditions in a focused and brief manner, offer scalability of industrial interest and a safer handling of the CO used in the reaction.

The use of continuous chemical technologies is growing in recent years both in industry^10^ and academia.^11^ This technique allows to automate processes, improves efficiency, and is generally more sustainable.^12^ Optimization of reaction conditions is faster, and the scaling up of the processes is facilitated. Safety improvements are significant especially when working with dangerous chemical substances or gases. The uniform mixing at the entrance of the reactor avoids the formation of hot spots in the reaction. In addition, it is easy to safely introduce defined amounts of gases into the system that mix better with solutions, which presents an advantage for the use of toxic or flammable gases such as hydrogen or CO.^13^

However, not all reactions find advantages when transferred to this methodology. The Hitchhiker's Guide has been published, describing the real benefits of employing the flow methodology.^14^ Solubility is a particular issue in flow methods. Substrates must enter the system dissolved through the pumps, thus avoiding any precipitation of substrates, intermediates or insoluble products which can cause clogging in the reactor.

Our research group has been working on the development of flow chemistry methodologies applied to the Pauson–Khand reaction^15^ and [2 + 2 + 2] cyclotrimerizations.^16^ Herein, we present a continuous process for the synthesis of amides through aminocarbonylation reactions of thiols.

## 2 Results and discussion

For the first optimization of the conditions, 4-methoxythiophenol and benzylamine/*p*-chlorobenzylamine were used as model substrates. These reactions were carried out in a stainless steel pressure tube. The effect of CO pressure, solvent, reagents equivalents, catalyst loading, temperature and reaction time were examined and a summary of the optimization study is shown in Table 1. Dioxane was selected as solvent for its coordinating ability. In addition to the desired amides (1 and 5), the main side products observed were the disulfide 2, and the ureas 3 and 6. Thioester 4 was detected but not isolated in most reactions. The table shows the yields of the desired amides and the two main side products.

**Table 1:** Optimization of reaction conditions of the aminocarbonylation^a^

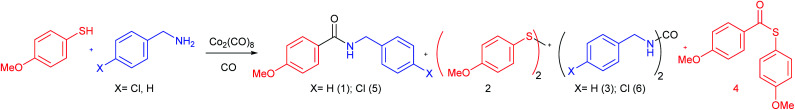										
No.	Solvent	*T* (°C)	CO (bar)	*t* (min)	Equiv. R-SH	Equiv. R′-NH_2_ (X)	Cat., equiv.	Yield (2)^b^ (%)	Yield (3/6)^b^ (%)	Yield (1/5)^b^ (%)
1	1,4-Dioxane	220	0	30	1	1	Co_2_(CO)_8_ 1.0	16	11	37
2	1,4-Dioxane	220	15	30	1	1	—	33	—	—
3	1,4-Dioxane	220	10	30	1	1	Co_2_(CO)_8_ 0.1	51	15	26
4	1,4-Dioxane	140	15	30	1	1	Co_2_(CO)_8_ 0.1	28	5	11
5	1,4-Dioxane	220	15	30	1	1	Co_2_(CO)_8_ 0.3	30	21	44
6	^*i*^PrOH	180	15	30	1	1	Co_2_(CO)_8_ 0.3	31	15	41
7	Toluene	220	15	30	1	1	Co_2_(CO)_8_ 0.3	30	15	52
8^c^	Toluene	220	15	60	2	1 (Cl)	Co_2_(CO)_8_ 0.3	34	5	58
9	Toluene	180	15	30	1	1	[Rh(CO)_2_]_2_ 0.3	24	66	n.i.^d^
10	Toluene	180	15	30	1	1	Ru_3_(CO)_12_ 0.3	—	—	—
11	Toluene	180	15	30	1	1	Mo(CO)_6_ 0.3^e^	68	20	n.i.^d^
12^f^	Tol., AcOH^g^	220	15	60	2	1 (Cl)	Co_2_(CO)_8_ 0.3	22	5^h^	69
13	AcOH	220	15	60	2	1 (Cl)	Co_2_(CO)_8_ 0.3	29	45^h^	n.i.^d^

a All these reactions were carried out in a concentration of 0.25 M.

b In pure product.

c Method A.

d n. i.: isolation was not performed, yield was estimated in less than 5% although product was detected in the crude mixture.

e 5 equiv. of DMSO were added.

f Method B.

g 1.0 equiv.

h *N*-Benzylacetamide.

Using a stoichiometric amount of the cobalt complex lead to the amide with a 37% yield together with the formation of the other secondary products (entry 1). The need to use the cobalt catalyst was verified (entry 2). It was also found that the best CO pressure was 15 bar and the best reaction temperature was 220 °C since, at lower pressure or temperature, yields in 1 were poor (entries 3 and 4). As the final product yield was still moderate, we increased the catalyst loading onto 30 mol%. The amide yield increased up to 44% (entry 5). Next we used isopropanol and toluene as solvents reaching better results with the latter (entries 6 and 7). As the main side process is the formation of disulfide 2, the initial amount of thiol was increased to 2.0 equiv. and the reaction time was set to 60 min, reaching, under these conditions, a 58% yield in 1 (entry 8). Next step was to check if the process could be mediated by other metal-carbonyl complexes. Both [Rh(CO)_2_Cl]_2_ and Mo(CO)_6_ (combined with DMSO as promoter) gave poor conversions in the amide, whereas the reaction with Ru_3_(CO)_12_ produce extensive decomposition of the reagents. At this point the best results were achieved with 2.0 equiv. of thiol, 1.0 equiv. of amine, 0.3 equiv. of Co_2_(CO)_8_, 1 hour of reaction, at 220 °C and with 15 bar of CO (method A, entry 8). In order to disfavour the formation of the disulfide we added variable amounts of acetic acid. In particular when adding 1 equiv. of this acid, the amide was obtained in a 69% yield (entry 12, method B). Compound 5 had been described in 48% yield through a palladium catalysed carbonylation of an aryl chloride.^17^ Addition of acetic acid, revealed useful with some of the examples studied below. Increasing the amount of acetic acid or using it as solvent did not produce any improvement but favoured the formation of the corresponding *N*-benzylacetamide instead of the desired amide (entry 13). With this method, no urea was formed. The role of the acid is not clear, but we assume it may avoid the formation of sulfides that could be involved in the formation of the disulfide 2.

**Fig. 1:**
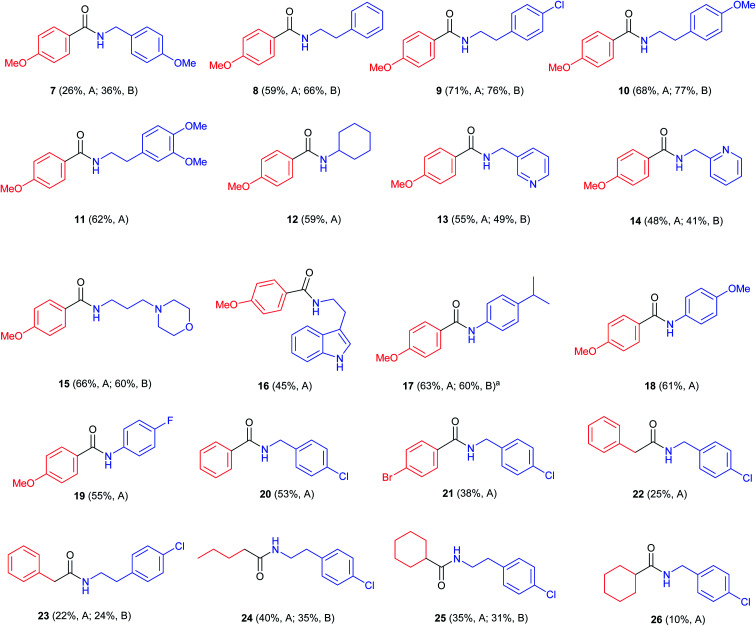
Study of the scope of the reaction on different aliphatic and aromatic amines. Reaction conditions: method A: 30 mol% Co_2_(CO)_8_, toluene, 220 °C, 15 bar CO, 60 min, 2.0 equiv. of thiol, 1.0 equiv. of amine, 0.3 equiv. catalyst, 15 bar CO, 220 °C. Method B: same as A, adding 1.0 equiv. of AcOH. ^*a*^When using 1.0 equiv. of TFA, yield was 41%.

With these two possible methods in hand we developed a scope study using different thiols and amines (Fig. 1).

First, *p*-methoxythiophenol, was reacted with different amines showing moderate results with 4-methoxybenzylamine (7; 36%), and good yields of the desired amides when combined with phenethylamines (8–11; 62–77%). Other aliphatic amines such as cyclohexylamine (12), pyridylmethylamine (13 and 14), 3-(*N*-morpholinyl)propylamine (15) and 2-(3-indolyl)ethylamine (16) gave the corresponding products in slightly lower yields (45–66%). In particular, compound 12 (59%) had been described by an oxidative amidation/transamidation in 54% yield.^18^ The reaction with anilines was also possible (17–19; 55–63%). It was found that the use of 1.0 equiv. of AcOH led to an improvement in yields of *N*-phenethylamides and *N*-benzylamides, but did not increase the yield of *N*-phenylamides. In case of the synthesis of compound 17 we tried adding a stronger acid, TFA, but this led to lower yields (41%). When other aromatic thiols were reacted with 4-chlorobenzylamine it was observed that yields decreased as the aromatic ring was more electron deficient (20, 21; 53% and 38% respectively). Strongly deactivated thiophenols such as *p*-nitrothiophenol did not react. When using aliphatic thiols, the final amides were isolated in low yields (22–26; 10–38%). As a whole, the best results were observed when using electron-rich aromatic thiols with aliphatic amines, in particular with phenethylamines. These thiols also react with anilines, but with a somewhat lower yield. On the other hand, the main limitation of the process is that aliphatic thiols react only with aliphatic amines and in low yields. This behaviour may be due to an increased tendency for the formation of disulfides.

The aminocarbonylation process was next transferred into a flow equipment in order to control the amount of CO gas used in the reaction and to try to reduce the formation of secondary products. This could render a safer procedure that could be scaled up allowing a possible multigram synthesis of amide containing drugs. Table 2 caption shows the configuration of the flow equipment used. The gas is introduced through a mass flow controller (MFC), and mixed with the solution of the starting substrates and the catalyst. The resulting mixture is pumped into a 20 mL reactor located inside an oven. Then, through a gas–liquid separator, the reaction gases (excess of CO and COS formed during the process) are released in a controlled manner. The main issue in the flow procedure was the solubility of the reaction mixture which was not complete in toluene. Thus, we used dioxane, but the system showed some clogging due to partial precipitation of the reaction products. After an extensive study with various solvents it was observed that all the components did dissolve in NMP, so we continued with this solvent and carried out a reaction in the steel tube for comparison, where the amide was obtained in 56% yield (entry 1, Table 2). This is similar to previous results in toluene. Next, a study of flow conditions was carried out, optimizing: pressure, temperature, reaction concentration, CO and catalyst equivalents. The first reaction was performed with a stoichiometric amount of the cobalt complex and no CO gas. This led to a 55% yield of the amide (entry 2), which improved the batch result (Table 1, entry 1, 37%). Next, it was found that 3 equiv. of CO were enough to achieve total conversion. Using a concentration of 0.25 M of the amine, at 220 °C, with 0.3 equiv. of metal complex, less amount of dimer was formed. No urea was detected in these reactions in flow. The residence time was 8.5 min. Under these conditions (method C, entry 4), the amide was isolated in 54% yield, slightly below the best batch result but giving a cleaner crude mixture. Attempts to lower the catalyst loading or the pressure or temperature led to worse results, possibly due to the increase in disulfide formation. No improvement was noted if increasing temperature to 240 °C and/or pressure. Finally, an injection was made using 1.0 equiv. of AcOH which led to obtaining amide 5 in a 56% yield (method D). Using this latter method, a long run reaction was performed using 1 g of amine, which allowed the synthesis of the amide in 55% yield, after 52 min of total run time. With this flow methodology it is accessible to obtain these products in a safer way, since it is possible to control the amount of CO gas that enters and leaves the system.

**Table 2:** Summary of the screening of conditions done in a flow chemistry reactor^a^

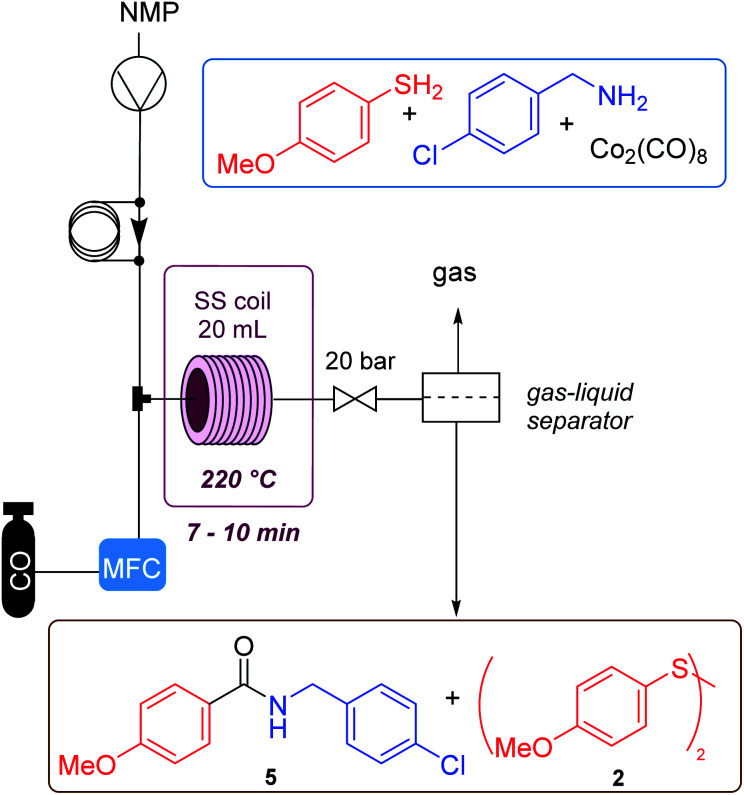								
No.	Conc. (M)	Press. (bar)	*T* (°C)	Equiv. CO	Equiv. Cat.	R. t. (min)	Ratio 5 : 2^b^	Yield^c^5 (%)
1^d^	0.25	15	220		0.3	60	60 : 40	56
2	0.25	20	220	0	1	8	60 : 40	55
3	0.25	20	220	5	0.3	8.5	48 : 52	45
4^e^	0.25	20	220	3	0.3	8.5	53 : 47	54
5	0.1	20	220	3	0.3	7	48 : 52	n.i.^f^
6	0.5	20	220	3	0.3	8	48 : 52	48
7	0.25	20	220	3	0.15	7	31 : 69	n.i.^f^
8	0.25	28	220	3	0.3	9	45 : 55	n.i.^f^
9	0.25	10	220	3	0.3	6	31 : 69	n.i.^f^
10	0.25	20	240	3	0.3	7	43 : 57	n.i.^f^
11	0.25	20	200	3	0.3	7	40 : 60	n.i.^f^
12^g^	0.25	20	220	3	0.3	7.5	57 : 43	56
13^h^	0.25	20	220	3	0.3	8.5^i^	60 : 40	55

a All reactions were performed with 1.0 equiv. of the amine and 2.0 equiv. of thiol (method C).

b Ratio estimated based on the integrals of well resolved ^1^H NMR signals of the crude mixture.

c In pure product.

d Batch reaction.

e Method-C.

f Isolation was not done.

g Including 1.0 equiv. of AcOH (method D).

h Long run with 1 g of amine.

i 52 min of total operation time.

**Scheme 1:**
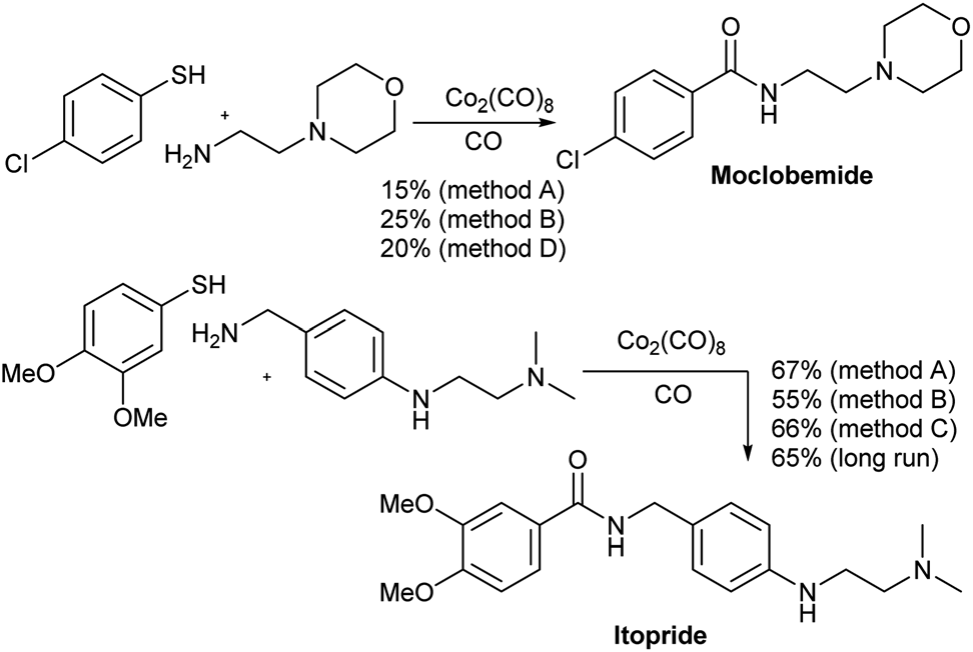
Synthesis of moclobemide and itopride.

The aminocarbonylation methods developed above were used in the continuous production of two drugs, namely moclobemide and itopride (Scheme 1). Moclobemide is used in the treatment of depression. Its mechanism of action consists of the reversible inhibition of MAO.^19^ On the other hand, itopride is used for the treatment of dyspepsia and other gastrointestinal disorders.^20^ In both cases, the reaction was first carried out in a sealed steel tube. Thus, moclobemide was isolated in 21 and 25% yield using methods A and B, respectively whereas itopride gave 67 and 55% yield, respectively, the same two methods. In these cases, the addition of 1.0 equiv. of AcOH gave better yielding only in the case of moclobemide. On the other hand, when the reaction was carried out in the flow equipment, the yield of moclobemide was 20% (method D) and that of itopride 66%, using method C. In the case of moclobemide, the poor result observed may be due to the fact that the starting thiol contains an electrowithdrawing group, which makes this aminocarbonylation run less favorable. Itopride was obtained in 65% yield when preforming a long run with 1 g of the starting amine. A total run time of 52 min was used for this reaction.

**Scheme 2:**
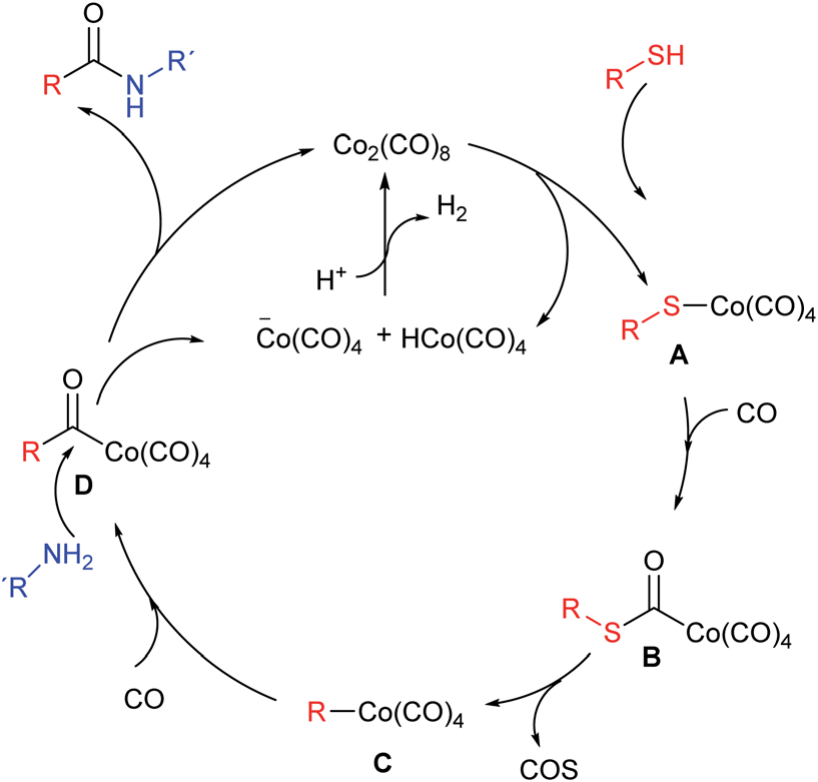
Reaction pathway for the aminocarbonylation of thiols.

The plausible mechanism of this reaction is outlined in Scheme 2. Following Alper's proposal,^5^ the thiol splits the cobalt carbonyl complex giving A, which incorporates a CO unit (B) and eliminates COS to produce intermediate C. A new CO insertion gives D which is attacked by the amine to yield the final amide plus one cobalt anionic species which, combined with the hydride released in the first step, regenerates the catalyst.

## 3 Experimental

### 3.1 General

All chemicals were obtained from Aldrich/Merck (St. Louis, MO, USA), VWR (Radnor, PA, USA) and Fluorochem (Derbyshire, UK). TLC analyses were performed on Merck silica gel 60 F254 plates using phosphomolybdic acid or anisaldehyde and heat for detection. Silica gel NORMASIL 60 40–63 μm was used for flash chromatography. NMR spectra were recorded on a Bruker spectrometer (400 MHz or 300 MHz for ^1^H and 100 MHz or 75 MHz for ^13^C). Chemical shifts are reported in *δ* ppm referenced to CDCl_3_ (*δ* = 7.26 for ^1^H and 77.00 for ^13^C). Infrared spectra were performed in a Perkin-Elmer spectrum 100 (Agilent, Santa Clara, CA, USA). Melting points of solid compounds were determined using a Stuart Scientific Melting Point Apparatus SMP3 (Stuart, Staffordshire, UK). Microanalyses were done on a LECO CHNS-932 (LECO, St. Joseph, MI, USA).

### 3.2 General procedure for batch protocols (methods A and B)

Batch protocol reactions were carried out in a 15 mL steel reactor, using 5.0 mL of nitrogen degassed dry toluene where 0.36 mmol (123 mg) of octacarbonyl dicobalt were dissolved and then, 1.2 mmol of the starting amine, and 2.4 mmol of the starting thiol. After sealing the reactor, it was saturated with 2–4 bar of carbon monoxide and evacuated three times, and then, filled with 15 bar of CO (Method A); additionally, 1.0 equiv. of AcOH was added (Method B). The reaction was kept stirring in an aluminium block at 220 °C for 1 hour. After that time, the reactor was cooled at room temperature in cool water, and the excess of pressure was released. After evaporating the reaction slurry at low pressure, the final product was purified through a silica column in gradient of hexane and EtOAc (from 4 : 1 to 1 : 1).

### 3.3 General procedure for flow protocols (methods C and D)

The flow system is a PFR (Plug flow reactor, tubular reactor, composed by a 316 stainless steel tube with internal diameter of 17 mm and 25 mm of external diameter, volume = 20 mL) in a forced air oven, with one feeding line with a semi-preparative HPLC pump ASI Model 501. The system pressure is automated, controlled by a high precision needle backpressure valve and WIKA pressure sensor. CO is introduced by a Bronkhorst mass flow controller calibrated for this gas, and it is mixed with the solution in a T-shape stainless steel piece. The system has a gas liquid separator after the reactor. Method C: injections in the flow system were carried out using NMP as solvent (0.25 M), 2.0 equiv. of the thiophenol, 1.0 equiv. of the amine, 0.3 equiv. of the catalyst, 3.0 equiv. of CO. The pumping flow rate was set at 1.5 mL min^−1^, the CO at 26 mL N min^−1^, and the system pressure was set at 20 bar. Method D: additionally, the mixture contained 1.0 equiv. of AcOH. NMP was removed by distillation at 90 °C and using a high-vacuum pump.

#### *N*-Benzyl-4-methoxybenzamide, (1)^21^

White solid, 150 mg, 52% (method A). ^1^H NMR (CDCl_3_) *δ* 7.76 (d, *J* = 8.9 Hz, 2H, 2× Ar–H), 7.36–7.26 (m, 5H, 5× Ar–H), 6.92 (d, *J* = 8.9 Hz, 2H, 2× Ar–H), 6.27 (s, 1H, NH), 4.64 (d, *J* = 5.7 Hz, 2H, CH_2_), 3.85 (s, 3H, OMe). In accordance to those data described in the literature.

#### *N*-(4-Chlorobenzyl)-4-methoxybenzamide, (5)^17^

White solid, 192.1 mg, 58% (method A), 228.5 mg, 69% (method B), 178.8 mg, 54% (method C), 185.5 mg, 56% (method D), 1071.7 mg, 55% (long run with 1 g of starting amine). ^1^H NMR (CDCl_3_) *δ* 7.76 (d, *J* = 8.8 Hz, 2H, 2× Ar–H), 7.33–7.27 (m, 4H, 4× Ar–H), 6.92 (d, *J* = 8.8 Hz, 2H, 2× Ar–H), 6.32 (s, 1H, NH), 4.61 (d, *J* = 5.8 Hz, 2H, CH_2_), 3.85 (s, 3H, OMe). In accordance to those data described in the literature.

#### 4-Methoxy-*N*-(4-methoxybenzyl)benzamide, (7)^22^

White solid, 84.5 mg, 26% (method A), 117.1 mg, 36% (method B) and 101.0 mg, 32% (method D). ^1^H NMR (CDCl_3_) *δ* 7.74 (d, *J* = 8.9 Hz, 2H, 2× Ar–H), 7.29 (d, *J* = 8.7 Hz, 2H, 2× Ar–H), 6.9 (t, *J* = 8.7 Hz, 4H, 3× Ar–H), 6.2 (s, 1H, NH), 4.57 (d, *J* = 5.5 Hz, 2H, CH_2_), 3.84 (s, 3H, OMe), 3.81 (s, 3H, OMe). In accordance to those data described in the literature.

#### 4-Methoxy-*N*-phenethylbenzamide, (8)^23^

White solid, 180.5 mg, 59% (method A), 202.0 mg, 66% (method B). ^1^H NMR (CDCl_3_) *δ* 7.65 (d, *J* = 8.8 Hz, 2H, 2× Ar–H), 7.35–7.31 (m, 2H, 2× Ph–H), 7.25–7.23 (m, 2H, 3× Ph–H), 6.90 (d, *J* = 8.8 Hz, 2H, 2× Ar–H), 6.02 (s, 1H, NH), 3.84 (s, 3H, OMe), 3.71 (q, *J* = 6.8 Hz, CH_2_N), 2.93 (t, *J* = 6.8 Hz, CH_2_Ph). In accordance to those data described in the literature.

#### *N*-(4-Chlorophenethyl)-4-methoxybenzamide, (9)

White solid, 247.1 mg, 71% (method A), 264.5 mg, 76% (method B). ^1^H NMR (CDCl_3_) *δ* 7.66 (d, *J* = 8.8 Hz, 2H, 2× Ar–H), 7.29 (d, *J* = 8.3 Hz, 2H, 2× Ar–H), 7.17 (d, *J* = 8.3 Hz, 2H, 2× Ar–H), 6.91 (d, *J* = 8.8 Hz, 2H, 2× Ar–H), 6.06 (s, 1H, NH), 3.84 (s, 3H, OMe), 3.68 (q, *J* = 6.7 Hz, 2H, CH_2_N), 2.90 (t, *J* = 6.7 Hz, 2H, CH_2_Ar). ^13^C NMR (CDCl_3_) *δ* 167.0 (CO), 162.2 (C–Ar), 137.5 (C–Ar), 132.3 (C–Ar), 130.2 (2× CH–Ar), 128.8 (2× CH–Ar), 128.6 (2× CH–Ar), 126.7 (C–Ar), 113.8 (2× CH–Ar), 55.4 (OMe), 41.0 (CH_2_), 35.2 (CH_2_). IR (NaCl, cm^−1^): 3355, 1638, 1538. Anal. calcd for C_16_H_16_ClNO_2_ calc.: C, 66.32; H, 5.57; N, 4.83. Found: C, 66.27; H, 5.37; N, 4.72. Mp 188–190 °C.

#### *N*-(4-Methoxyphenethyl)-4-methoxybenzamide, (10)

Pale yellow solid, 68% 232.6 mg, (method A) and 263.3 mg, 77% (method B). ^1^H NMR (CDCl_3_) *δ* 7.65 (d, *J* = 8.8 Hz, 2H, 2× Ar–H), 7.15 (d, *J* = 8.5 Hz, 2H, 2× Ar–H), 6.90 (d, *J* = 8.8 Hz, 2H, 2× Ar–H), 6.87 (d, *J* = 8.5 Hz, 2H, 2× Ar–H), 6.01 (s, 1H, NH), 3.84 (s, 3H, OMe), 3.80 (s, 3H, OMe), 3.67 (q, *J* = 6.7 Hz, 2H, CH_2_N), 2.87 (t, *J* = 6.8 Hz, 2H, CH_2_Ar). ^13^C NMR (CDCl_3_) *δ* 167.0 (CO), 162.1 (C–Ar), 158.3 (C–Ar), 131.0 (C–Ar), 129.8 (2× CH–Ar), 128.6 (2× CH–Ar), 126.9 (C–Ar), 114.1 (2× CH–Ar), 113.7 (2× CH–Ar), 55.4 (OMe), 55.3 (OMe), 41.3 (CH_2_), 34.9 (CH_2_). IR (NaCl, cm^−1^): 3350, 1638, 1542. Anal. calcd for C_17_H_19_NO_3_ calc.: C, 71.56; H, 6.71; N, 4.91. Found: C, 71.84; H, 6.81; N, 4.79. Mp 145–147 °C.

#### *N*-(3,4-Dimethoxyphenethyl)-4-methoxybenzamide, (11)^24^

White solid, 234.4 mg, 62% (method A). ^1^H NMR (CDCl_3_) *δ* 7.66 (d, *J* = 8.7 Hz, 2H, 2× Ar–H), 6.90 (d, *J* = 8.7 Hz, 2H, 2× Ar–H), 6.83 (d, *J* = 7.9 Hz, 1H, Ar–H), 6.77 (dd, *J* = 8.0, 1.9 Hz, 1H, Ar–H), 6.75 (d, *J* = 1.8 Hz, 1H, Ar–H) 6.02 (s, 1H, NH), 3.87 (s, 3H, OMe), 3.84 (s, 3H, OMe), 3.839 (s, 3H, OMe), 3.68 (q, *J* = 6.7 Hz, 2H, CH_2_N), 2.87 (t, *J* = 6.8 Hz, 2H, CH_2_Ar). In accordance to those data described in the literature.

#### *N*-Cyclohexyl-4-methoxybenzamide, (12)^18^

White solid, 165 mg, 59% (method A). ^1^H NMR (CDCl_3_) *δ* 7.71 (d, *J* = 8.8 Hz, 2H, 2× Ar–H), 6.91 (d, *J* = 8.8 Hz, 2H, 2× Ar–H), 5.84 (d, *J* = 5.5 Hz, 1H, NH), 4.02–3.92 (m, 1H, CH), 3.84 (s, 3H, OMe), 2.05–2.00 (m, 2H, Cy), 1.77–1.64 (m, 3H, Cy), 1.49–1.38 (m, 2H, Cy), 1.27–1.06 (m, 3H, Cy). In accordance to those data described in the literature.

#### 4-Methoxy-*N*-(pyridin-3-ylmethyl)benzamide, (13)

White solid, 159.7 mg, 55% (method A) and 142.3 mg, 49% (method B). ^1^H NMR (CDCl_3_) *δ* 8.62 (s, 1H, Py–H), 8.56 (s, 1H, Py–H), 7.76 (d, *J* = 9.1 Hz, 2H, 2× Ar–H), 7.71 (d, *J* = 8.1 Hz, 1H, Py–H), 7.30–7.21 (m, 1H, Py–H), 6.93 (d, *J* = 9.1 Hz, 2H, 2× Ar–H), 6.85 (s, 1H, Py–H), 6.43 (s, 1H, NH), 4.66 (d, *J* = 6.0 Hz, 2H, CH_2_), 3.85 (s, 3H, OMe). ^13^C NMR (CDCl_3_) *δ* 167.1 (CO), 162.3 (C–Ar), 149.1 (CH Py), 148.8 (CH Py), 135.7 (CH Py), 134.3 (C Ar or Py), 128.8 (2× CH–Ar), 126.2 (C Ar or Py), 123.6 (CH Py), 113.8 (2× CH–Ar), 55.3 (OMe), 41.4 (CH_2_). IR (NaCl, cm^−1^): 3432, 1639, 1607, 1545. Anal. calcd for C_14_H_14_N_2_O_2_ calc.: C, 69.41; H, 5.82; N, 11.56. Found: C, 69.26; H, 5.95; N, 11.62. Mp 154–156 °C.

#### 4-Methoxy-*N*-(pyridin-2-ylmethyl)benzamide, (14)^25^

White solid, 139.4 mg, 48% (method A) and 119.1 mg, 41% (method B). ^1^H NMR (CDCl_3_) *δ* 8.56 (d, *J* = 4.5 Hz, 1H, Py–H), 7.85 (d, *J* = 8.8 Hz, 2H, 2× Ar–H), 7.68 (td, *J* = 7.8, 1.8 Hz, 1H, Py–H), 7.48 (s, 1H, NH), 7.33 (d, *J* = 7.9 Hz, 1H, Py–H), 7.21 (dd, *J* = 3.8, 4.8 Hz, 1H, Py–H), 6.94 (d, *J* = 8.9 Hz, 2H, 2× Ar–H), 4.76 (d, *J* = 4.85 Hz, 2H, CH_2_), 3.86 (s, 3H, OMe). In accordance to those data described in the literature.

#### 4-Methoxy-*N*-(3-morpholinopropyl)benzamide, (15)

Pale yellow oil, 220.2 mg, 66% (method A) and 200.2 mg, 60% (method B). ^1^H NMR (CDCl_3_) *δ* 7.77 (d, *J* = 8.8 Hz, 2H, 2× Ar–H), 6.93 (d, *J* = 8.8 Hz, 2H, 2× Ar–H), 3.85 (s, 3H, OMe), 3.71 (t, *J* = 4.7 Hz, 4H, 2× CH_2_O), 3.85–3.54 (m, 2H, CH_2_N), 2.57–2.49 (m, 6H, 3× CH_2_N), 1.82–1.76 (m, 2H, C*H*_2_CH_2_N). ^13^C NMR (100 MHz, CDCl_3_) *δ* 166.9 (CO), 162.0 (C–Ar), 128.7 (2× CH–Ar), 127.2 (C–Ar), 113.6 (2× CH–Ar), 67.0 (2× CH_2_O), 58.7 (CH_2_N), 55.4 (OMe), 53.9 (2× CH_2_N), 40.5 (CH_2_NCO), 24.3 (*C*H_2_CH_2_N). Pale yellow oil. IR (NaCl, cm^−1^): 3460, 1633, 1607, 1549. Anal. calcd for C_15_H_22_N_2_O_3_ calc.: C, 64.73; H, 7.97; N, 10.06. Found: C, 64.95; H, 8.11; N, 10.22.

#### *N*-(2-(1*H*-indol-3-yl)ethyl)-4-methoxybenzamide, (16)^7^

White solid, 158.8 mg, 45% (method A). ^1^H NMR (CDCl_3_) *δ* 8.03 (s, 1H, NH), 7.68–7.60 (m, 3H, 3× Ar–H), 7.39 (dt, *J* = 8.2, 0.9 Hz, 1H, Ar–H), 7.25–7.19 (m, 1H, Ar–H), 7.14 (ddd, *J* = 8.0, 7.1, 1.0 Hz, 1H, Ar–H), 7.08 (d, *J* = 2.3 Hz, 1H, Ar–H), 6.87 (d, *J* = 8.5 Hz, 2H, 2× Ar–H), 6.10 (s, 1H, NH), 3.83 (s, 3H, OMe), 3.80 (q, *J* = 5.9 Hz, 2H, CH_2_N), 3.10 (t, *J* = 6.3 Hz, 2H, CH_2_Ar). In accordance to those data described in the literature.

#### *N*-(4-Isopropylphenyl)-4-methoxybenzamide, (17)

Brown solid, 203.4 mg, 63% (method A), 193.7 mg, 60% (method B), 41% (method C). ^1^H NMR (CDCl_3_) *δ* 7.85 (d, *J* = 8.6 Hz, 2H, 2× Ar–H), 7.69 (s, 1H, NH), 7.53 (d, *J* = 8.4 Hz, 2H, 2× Ar–H), 7.22 (d, *J* = 8.4 Hz, 2H, 2× Ar–H), 6.97 (d, *J* = 8.6 Hz, 2H, 2× Ar–H), 3.87 (s, 3H, OMe), 2.90 (septet, *J* = 6.9 Hz, CHMe_2_), 1.25 (d, 6H, *J* = 6.9, 2× CH_3_). ^13^C NMR (CDCl_3_) *δ* 165.2 (CO), 162.4 (C–Ar), 145.1 (C–Ar), 135.8 (C–Ar), 128.9 (2× CH–Ar), 127.3 (C–Ar), 127.0 (2× CH–Ar), 120.3 (2× CH–Ar), 114.0 (2× CH–Ar), 55.5 (CH_3_), 33.6 (CH), 24.0 (2× CH_3_). IR (NaCl, cm^−1^): 3320, 1646, 1517. Anal. calcd for C_17_H_19_NO_2_ calc.: C, 75.81; H, 7.11; N, 5.20. Found: C, 75.65; H, 7.18; N, 5.32. Mp 168–171 °C.

#### *N*-(4-Methoxyphenyl)4-methoxybenzamide, (18)^26^

White solid, 188.1 mg, 61% (method A). ^1^H NMR (CDCl_3_) *δ* 7.83 (d, *J* = 9.0 Hz, 2H, 2× Ar–H), 7.63 (s, 1H, NH), 7.52 (d, *J* = 8.4 Hz, 2H, 2× Ar–H), 6.97 (d, *J* = 9.5 Hz, 2H, 2× Ar–H), 6.91 (d, *J* = 8.9 Hz, 2H, 2× Ar–H), 3.87 (s, 3H, OMe), 3.81 (s, 3H, OMe). In accordance to those data described in the literature.

#### *N*-(4-Fluorophenyl)4-methoxybenzamide, (19)^27^

White solid, 161.7 mg, 55% (method A). ^1^H NMR (CDCl_3_) *δ* 7.84 (d, 2H, *J* = 8.9 Hz, 2× Ar–H), 7.70 (s, 1H, NH), 7.59 (dd, 2H, *J* = 5.0, 4.5 Hz), 7.07 (t, 2H, *J* = 8.4, 2H), 6.99 (d, 2H, *J* = 8.9 Hz, 2× Ar–H), 3.88 (s, 3H, OMe). In accordance to those data described in the literature.

#### *N*-(4-Chlorobenzyl)benzamide, (20)^28^

White solid, 156.4 mg, 53% (method A). ^1^H NMR (CDCl_3_) *δ* 7.80–7.77 (m, 2H, 2× Ar–H), 7.54–7.41 (m, 2H, 2× Ar–H), 7.37–7.28 (m, 4H, 4× Ar–H), 6.39 (s, 1H, NH), 4.62 (d, *J* = 5.8, 2H, CH_2_). In accordance to those data described in the literature.

#### 4-Bromo-*N*-(4-chlorobenzyl)benzamide, (21)

White solid, 147.7 mg, 38% (method A). ^1^H NMR (CDCl_3_) *δ* 7.66 (d, *J* = 8.7 Hz, 2H, 2× Ar–H), 7.58 (d, *J* = 8.7 Hz, 2H, 2× Ar–H), 7.33–7.27 (m, 4H, 4× Ar–H), 6.35 (s, 1H, NH), 4.61 (d, *J* = 5.8 Hz, 2H, CH_2_). ^13^C NMR (CDCl_3_) *δ* 166.5 (CO), 136.5 (C–Ar), 133.5 (C–Ar), 133.0 (C–Ar), 131.9 (2× CH–Ar), 129.2 (2× CH–Ar), 129.0 (2× CH–Ar), 128.6 (2× CH–Ar), 126.4 (C–Ar), 43.5 (CH_2_). IR (NaCl, cm^−1^): 3320, 1639, 1542. Anal. calcd for C_14_H_11_BrClNO calc.: C, 51.80; H, 3.42; N, 4.32. Found: C, 51.92; H, 3.49; N, 4.46. Mp 128–130 °C.

#### *N*-(4-Chlorobenzyl)-2-phenylacetamide, (22)^29^

White solid, 78.0 mg, 25% (method A). ^1^H NMR (CDCl_3_) *δ* 7.38–7.27 (m, 5H, 5× Ar–H), 7.25–7.24 (m, 2H, 2× Ar–H), 7.10 (d, *J* = 8.6 Hz, 2H, 2× Ar–H), 5.66 (s, 1H, NH), 4.37 (d, *J* = 6.0 Hz, 2H, CH_2_N), 3.63 (s, 2H, CH_2_Ph). In accordance to those data described in the literature.

#### *N*-(4-Chlorophenethyl)-2-phenylacetamide, (23)^30^

White solid, 72.3 mg, 22% (method A) and 78.9 mg, 24% (method B). ^1^H NMR (CDCl_3_) *δ* 7.35–7.29 (m, 3H, 3× Ar–H), 7.20–7.15 (m, 4H, 4× Ar–H), 6.94 (d, *J* = 8.5 Hz, 2H, 2× Ar–H), 5.28 (s, 1H, NH), 3.53 (s, 2H, CH_2_Ph), 3.43 (dd, *J* = 6.1, 6.7 Hz, 2H, CH_2_NCO), 2.69 (t, *J* = 7.0 Hz, 2H, CH_2_Ar). In accordance to those data described in the literature.

#### *N*-(4-Chlorophenethyl)pentanamide, (24)

White solid, 115.2 mg, 40% (method A) and 100.8 mg, 35% (method B). ^1^H NMR (CDCl_3_) *δ* 7.28 (d, *J* = 8.8 Hz, 2H, 2× Ar–H), 7.12, (d, *J* = 8.8 Hz, 2H, 2× Ar–H), 5.37 (s, 1H, NH), 3.49 (dd, *J* = 6.1, 6.0 Hz, 2H CH_2_NCO), 2.79 (t, *J* = 6.9 Hz, 2H, CH_2_Ar), 2.12 (t, *J* = 7.7 Hz, 2H, CH_2_CO), 1.61–1.53 (m, 2H, CH_2_), 1.36–1.25 (m, 2H, CH_2_), 0.89 (t, *J* = 7.5 Hz, 3H, CH_3_). ^13^C NMR (CDCl_3_) *δ* 173.1 (CO), 137.4 (C–Ar), 132.3 (C–Ar), 130.1 (2× CH–Ar), 128.7 (2× CH–Ar), 40.39 (CH_2_), 36.5 (CH_2_), 35.1 (CH_2_), 27.8 (CH_2_), 22.4 (CH_2_), 13.8 (CH_3_). IR (NaCl, cm^−1^): 3300, 1637, 1546. Anal. calcd for C_13_H_18_ClNO calc.: C, 65.13; H,7.57; N, 5.84. Found: C, 65.27; H, 7.35; N, 5.97. Mp. 67–69 °C.

#### *N*-(4-Chlorophenethyl)cyclohexanecarboxamide, (25)

White solid, 111.7 mg, 35% (method A) and 98.9 mg, 31% (method B). ^1^H NMR (CDCl_3_) *δ* 7.28 (d, *J* = 8.5 Hz, 2H, 2× Ar–H), 7.11 (d, *J* = 8.4 Hz, 2H, 2× Ar–H), 5.39 (s, 1H, NH), 3.48 (dd, *J* = 12.9, 6.9 Hz, 2H, CH_2_NCO), 2.78 (t, *J* = 6.9 Hz, 2H, CH_2_Ar), 2.00 (tt, *J* = 11.6, 3.3 Hz, 1H, CH), 1.81–1.75 (m, 4H, Cy), 1.67–1.62 (m, 1H, Cy), 1.43–1.33 (m, 2H, Cy), 1.29–1.17 (m, 3H, Cy). ^13^C NMR (CDCl_3_) *δ* 176.1 (CO), 137.5 (C–Ar), 132.3 (C–Ar), 130.1 (2× CH–Ar), 128.7 (2× CH–Ar), 45.5 (CH), 40.3 (CH_2_), 35.1 (CH_2_), 29.7 (2× CH_2_), 25.7 (3× CH_2_). IR (NaCl, cm^−1^): 3289, 1636, 1546. Anal. calcd for C_15_H_20_ClNO calc.: C, 67.79; H, 7.59; N, 5.27. Found: C, 67.68; H, 7.61; N, 5.36. Mp 132–134 °C.

#### *N*-(4-Chlorobenzyl)cyclohexanecarboxamide, (26)

Pale brown solid, 30.2 mg, 10% (method A). ^1^H NMR (CDCl_3_) *δ* 7.29 (d, *J* = 8.4 Hz, 2H, 2× Ar–H), 7.19 (s, *J* = 8.4 Hz, 2H, 2× Ar–H), 5.74 (s, 1H, NH), 4.40 (d, *J* = 5.8 Hz, 2H, CH_2_N), 2.11 (tt, *J* = 11.7, 3.7 Hz, 1H, CH), 1.90–1.86 (m, 2H, Cy), 1.82–1.78 (m, 2H, Cy), 1.68–1.63 (m, 2H, Cy), 1.46 (qd, *J* = 12.7, 3.3 Hz, 2H, Cy), 1.29–1.22 (m, 2H, Cy). ^13^C NMR (CDCl_3_) *δ* 175.9 (CO), 137.1 (C–Ar), 133.2 (C–Ar), 129.0 (2× CH–Ar), 128.8 (2× CH–Ar), 45.5 (CH), 42.6 (CH_2_N), 29.7 (2× CH_2_), 25.7 (3× CH_2_). IR (NaCl, cm^−1^): 3289, 1639, 1550. Anal. calcd for C_14_H_18_ClNO calc.: C, 66.79; H, 7.21; N, 5.56. Found: C, 66.91; H, 7.34; N, 5.69. Mp 128–130 °C.

#### 4-Chloro-*N*-(2-morpholinoethyl)benzamide (moclobemide)^31^

White solid, 67.8 mg, 21% (method A), 80.7 mg, 25% (method B) and 64.7 mg, 20% (method D). ^1^H NMR (CDCl_3_) *δ* 7.72 (d, *J* = 8.4 Hz, 2H, 2× Ar–H), 7.42 (d, *J* = 8.4 Hz, 2H, 2× Ar–H), 6.75 (s, 1H, NH), 3.75–3.70 (m, 4H, 2× OCH_2_), 3.55 (dd, *J* = 11.0, 5.6 Hz, 2H, CH_2_), 2.61 (t, *J* = 5.9 Hz, 2H, CH_2_), 2.51 (s, 4H, 2× CH_2_). In accordance to those data described in the literature.

#### *N*-(4-((2-(Dimethylamino)ethyl)amino)benzyl)-3,4-dimetho-xybenzamide (itopride)^32^

Pale red solid, 287.0 mg, 67% (method A), 253.6 mg, 55% (method B) and 282.7 mg, 66% (method C). ^1^H NMR (CDCl_3_) *δ* 7.44 (d, *J* = 1.9 Hz, 1H, Ar–H), 7.29–7.25 (m, 3H, 3× Ar–H), 6.89 (d, *J* = 8.6 Hz, 2H, 2× Ar–H), 6.83 (d, *J* = 8.4 Hz, 1H, Ar–H), 6.33 (s, 1H, NH), 4.55 (d, *J* = 5.6 Hz, 2H, PhCH_2_), 4.05 (t, *J* = 5.7 Hz, 2H, CH_2_), 3.91 (d, *J* = 3.3 Hz, 3H, OMe), 3.90 (s, 3H, OMe), 2.72 (t, *J* = 5.7 Hz, 2H, CH_2_), 2.33 (s, 6H, 2× CH_3_). In accordance to those data described in the literature.

## 4 Conclusions

A new methodology has been developed for the synthesis of amides from thiols and amines in which carbonyl is incorporated from CO. After studying all the reaction parameters, a methodology has been described that allows these amides to be obtained with variable yields. Specifically, it has been concluded that this synthesis occurs preferentially when aromatic thiols with electron-rich rings are used. On the other hand, the use of aliphatic thiols has given rise to reactions with low yields. In addition, various side reactions have been observed that compete with the formation of the amide, especially the formation of disulfides. A flow chemistry procedure has also been optimized to obtain these amides. In this procedure, the yields are similar than those obtained in a sealed tube while it allows better and safer control of the CO input. A long run experiment has shown the scalability of the process. The methodology developed has been applied to the synthesis of two drugs, moclobemide and itopride. The results have been very discreet in the first case and good in the second.

## Author contributions

Conceptualization of the work, J. P. -C. and G. D.; methodology, all authors; synthetic work, J. O.; writing—original draft preparation, J. P. -C.; writing—review and editing, all authors; and funding acquisition, J. P. -C. All authors have read and agreed to the published version of the manuscript.

## Conflicts of interest

There are no conflicts to declare.

## Supplementary Material

RA-011-D1RA04736A-s001

## References

[cit1] Wang X. (2019). Nat. Catal..

[cit2] Mancuso R., Della Ca' N., Veltri L., Ziccarelli I., Gabriele B. (2019). Catalysts.

[cit3] Palinkas N., Mikle G., Aranyi A., Peter A., Kollar L. (2020). ChemistrySelect.

[cit4] Yuan S., Han H., Li Y., Wu X., Bao X., Gu Z., Xia J. (2019). Angew. Chem., Int. Ed..

[cit5] Antebi S., Alper H. (1986). Can. J. Chem..

[cit6] Yan D.-M., Crudden C. M., Chen J.-R., Xiao W.-J. (2019). ACS Catal..

[cit7] Veatch A. M., Alexanian E. J. (2020). Chem. Sci..

[cit8] Sargent B. T., Alexanian E. J. (2019). Angew. Chem., Int. Ed..

[cit9] Cheruku S., M Sajith A., Narayana Y., Shetty P., Nagarakere S. C., Sagar K. S., Manikyanally K. N., Rangappa K. S., Mantelingu K. (2021). J. Org. Chem..

[cit10] Baumann M., Moody T. S., Smyth M., Wharry S. (2020). Org. Process Res. Dev..

[cit11] Domokos A., Nagy B., Szilágyi B., Marosi G., Nagy Z. K. (2021). Org. Process Res. Dev..

[cit12] Colella M., Carlucci C., Luisi R. (2018). Top. Curr. Chem..

[cit13] Mallia C. J., Baxendale I. R. (2016). Org. Process Res. Dev..

[cit14] Plutschack M. B., Pieber B., Gilmore K., Seeberger P. H. (2017). Chem. Rev..

[cit15] García-Lacuna J., Domínguez G., Blanco-Urgoiti J., Pérez-Castells J. (2017). Chem. Commun..

[cit16] García-Lacuna J., Domínguez G., Blanco-Urgoiti J., Pérez-Castells J. (2018). Org. Lett..

[cit17] Lagueux-Tremblay P. L., Fabrikant A., Arndtsen B. A. (2018). ACS Catal..

[cit18] Nandi J., Vaughan M. Z., Sandoval A. L., Paolillo J. M., Leadbeater N. E. (2020). J. Org. Chem..

[cit19] Fisar Z., Hroudová J., Raboch J. (2010). Neuroendocrinol. Lett..

[cit20] Huang X., Lv B., Zhang S., Fan Y. H., Meng L. N. (2012). World J. Gastroenterol..

[cit21] Achar T. K., Mal P. (2015). J. Org. Chem..

[cit22] Manasa K. L., Tangella Y., Krishna N. H., Alvala M. (2019). Beilstein J. Org. Chem..

[cit23] Xing D., Xu X., Yang L. (2009). Synthesis.

[cit24] Jia X., Yang D., Zhang S., Cheng J. (2009). Org. Lett..

[cit25] Fu L., Ying J., Wu X. (2019). J. Org. Chem..

[cit26] Yao Y., Zhao G., Hamze A., Alami M., Provot O. (2020). Eur. J. Org. Chem..

[cit27] Xu M., Zhang X., Shao Y., Han J., Zhong P. (2012). Adv. Synth. Catal..

[cit28] Karthik S., Muthuvel K., Gandhi T. (2019). J. Org. Chem..

[cit29] Ghosh S. C., Hong S. H. (2010). Eur. J. Org. Chem..

[cit30] Gruen A., Milen M., Foeldesi T., Abranyi-Balogh P., Drahos L., Keglevich G. (2013). Synth. Commun..

[cit31] Papadopoulos G. N., Kokotos C. G. (2016). J. Org. Chem..

[cit32] Hermange P., Lindhardt A. T., Taaning R. H., Bjerglund K., Lupp D., Skrydstrup T. (2011). J. Am. Chem. Soc..

