# Supplementary material for: Cobalt catalysed aminocarbonylation of thiols in batch and flow for the preparation of amides
Source: RSC Adv. 2021 Sep 13;11(48):30398–406. doi: 10.1039/d1ra04736a (PMC9041104; doi:10.1039/d1ra04736a)

Electronic Supplementary Material (ESI) for RSC Advances.
This journal is © The Royal Society of Chemistry 2021

ELECTRONIC SUPLEMENTARY INFORMATION

Cobalt catalysed aminocarbonylation of thiols in batch and flow for the preparation of amides

Jose Maria Orduña, Gema Domínguez and Javier Pérez Castells*

*N-benzyl-4-methoxybenzamide, (**1**).*

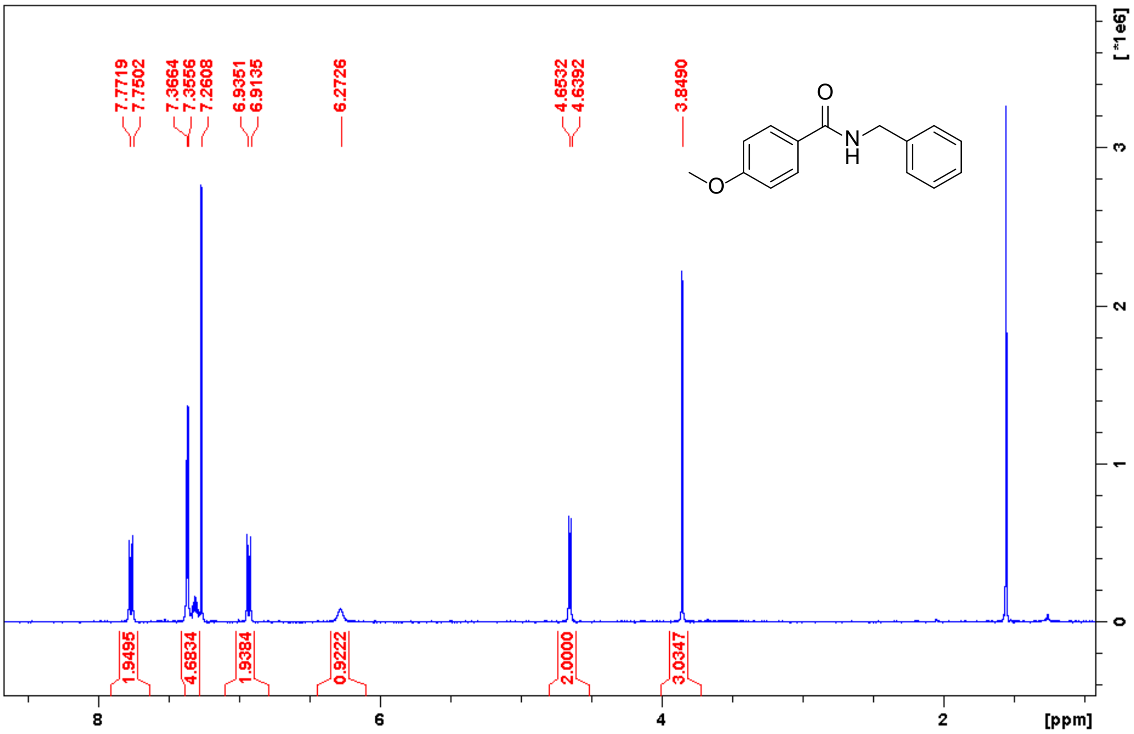

*N-(4-chlorobenzyl)-4-methoxybenzamide, (**5**).*

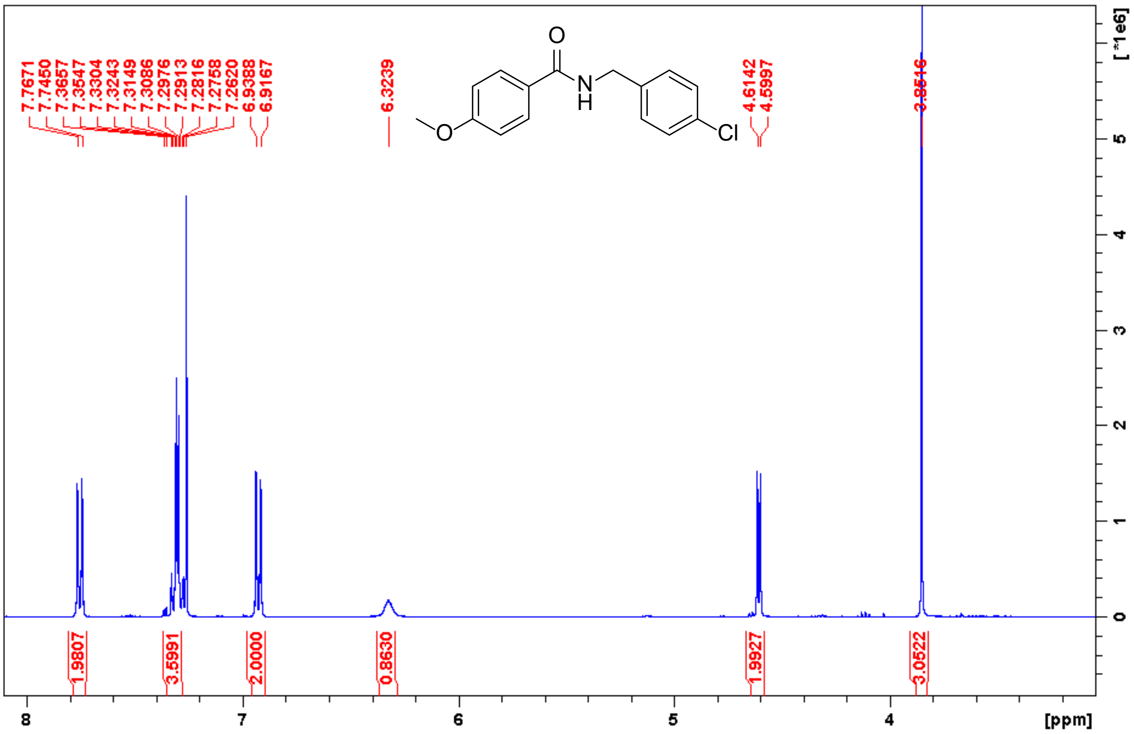

*4-methoxy-N-(4-methoxybenzyl)benzamide, (**7**).*

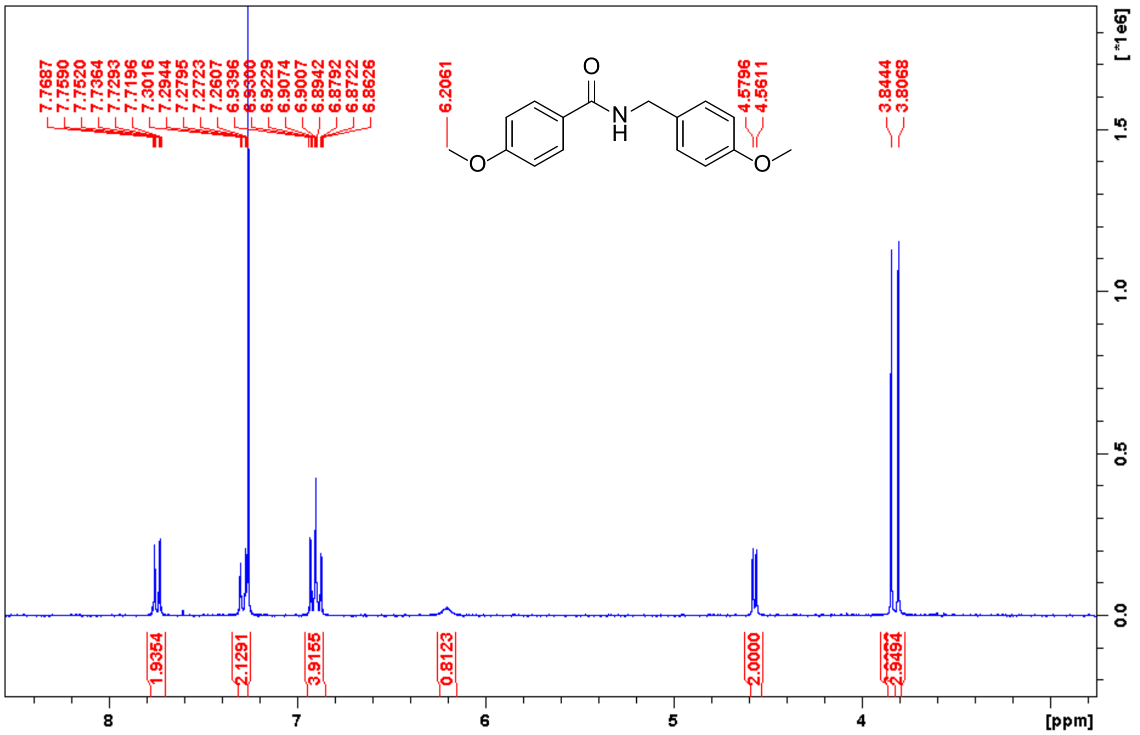

*4-methoxy-N-phenethylbenzamide, (**8**).*

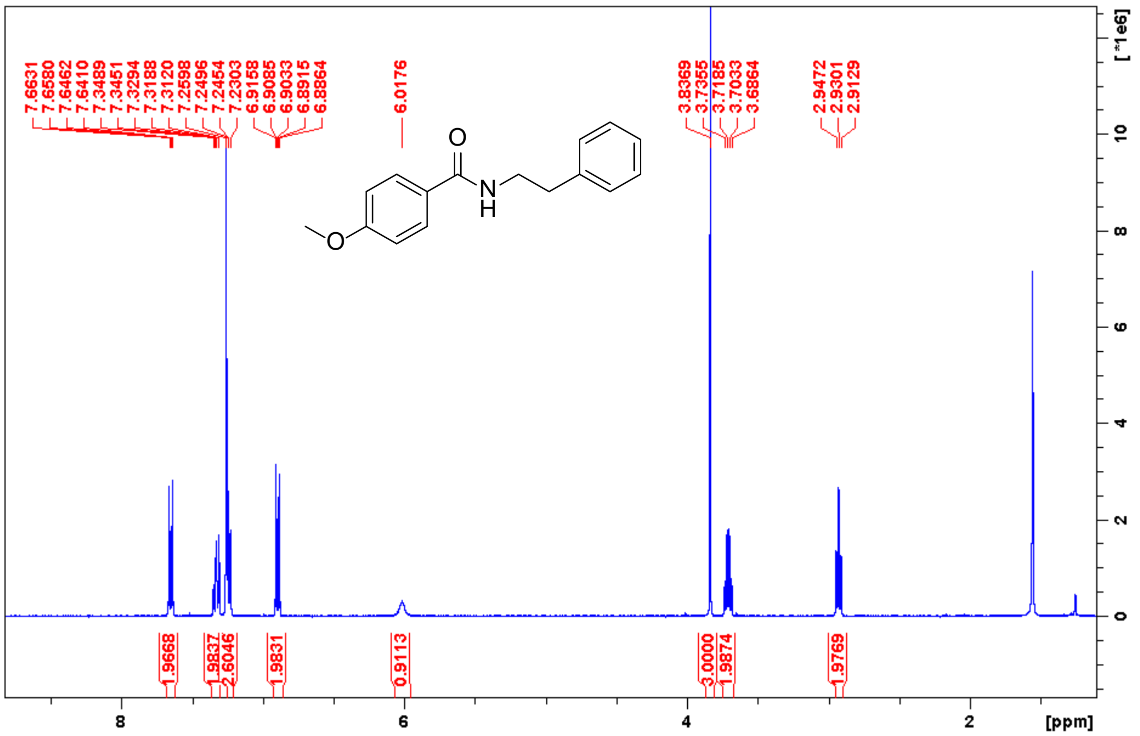

*N-(4-chlorophenethyl)-4-methoxybenzamide, (**9**).*

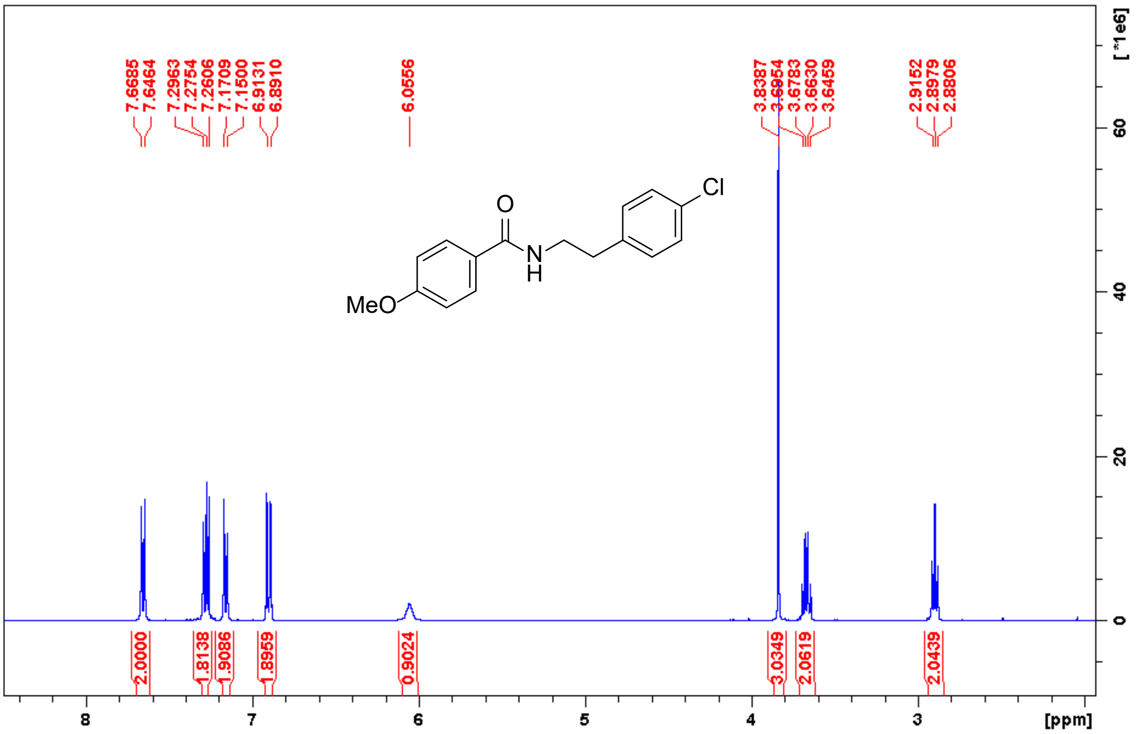

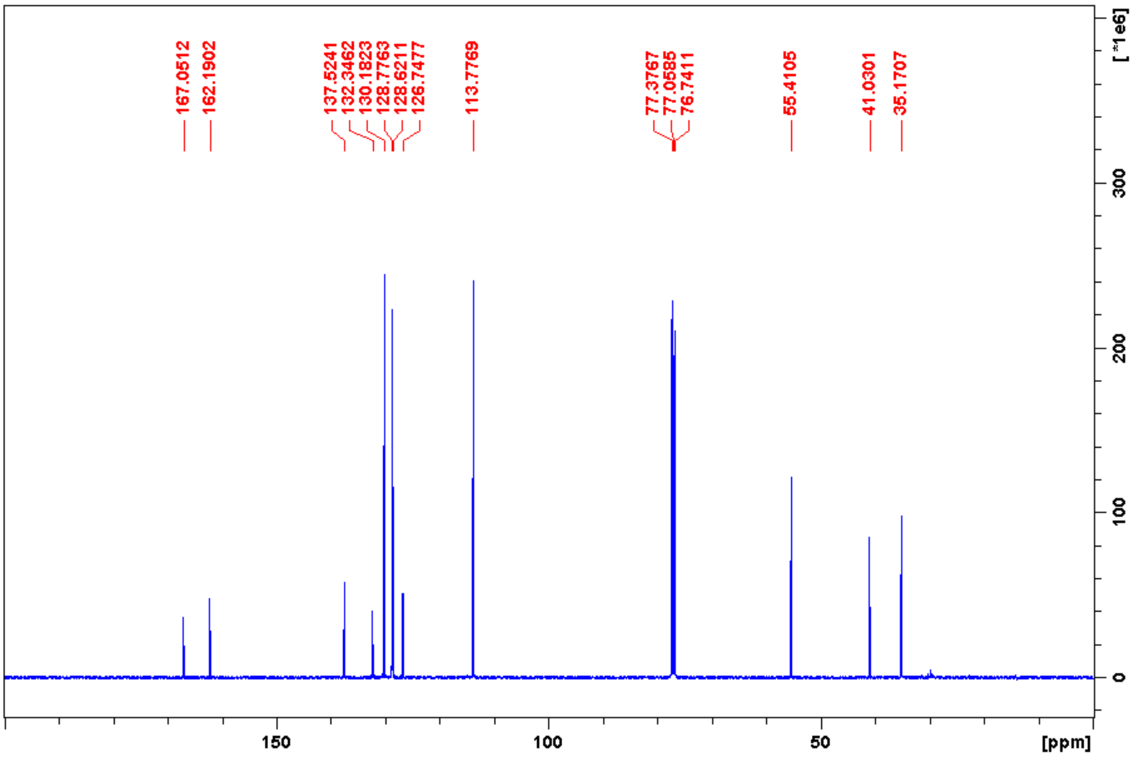

*N-(4-methoxyphenethyl)-4-methoxybenzamide, (**10**)*

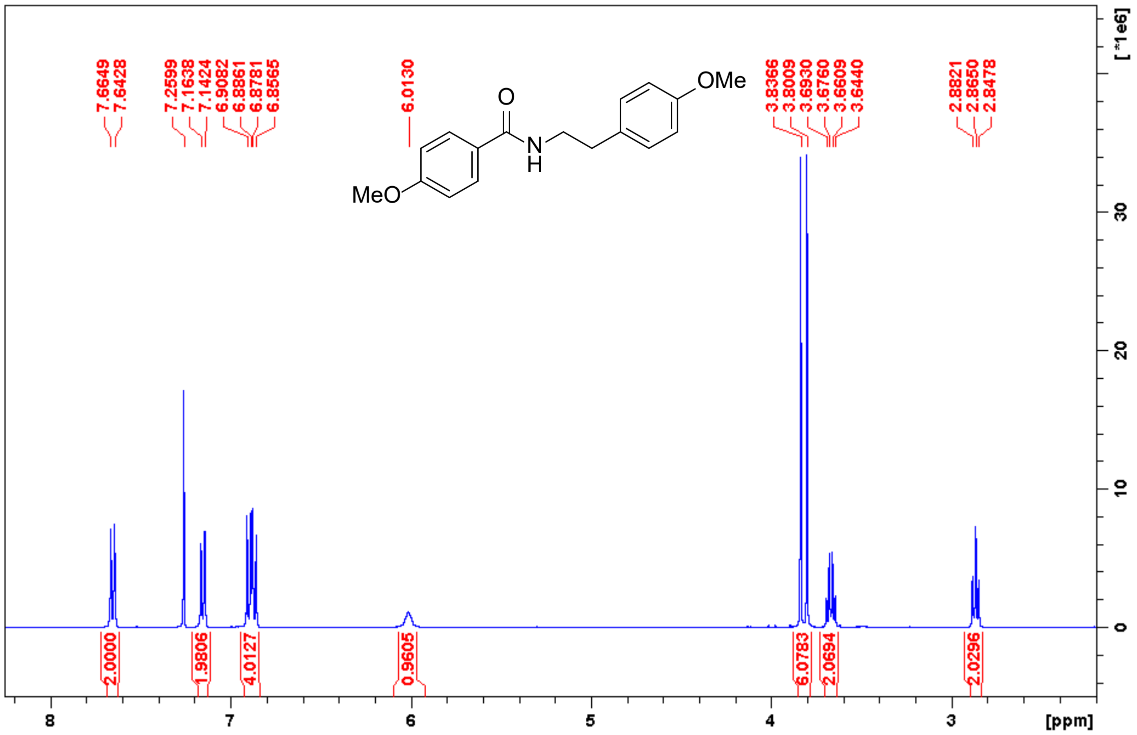

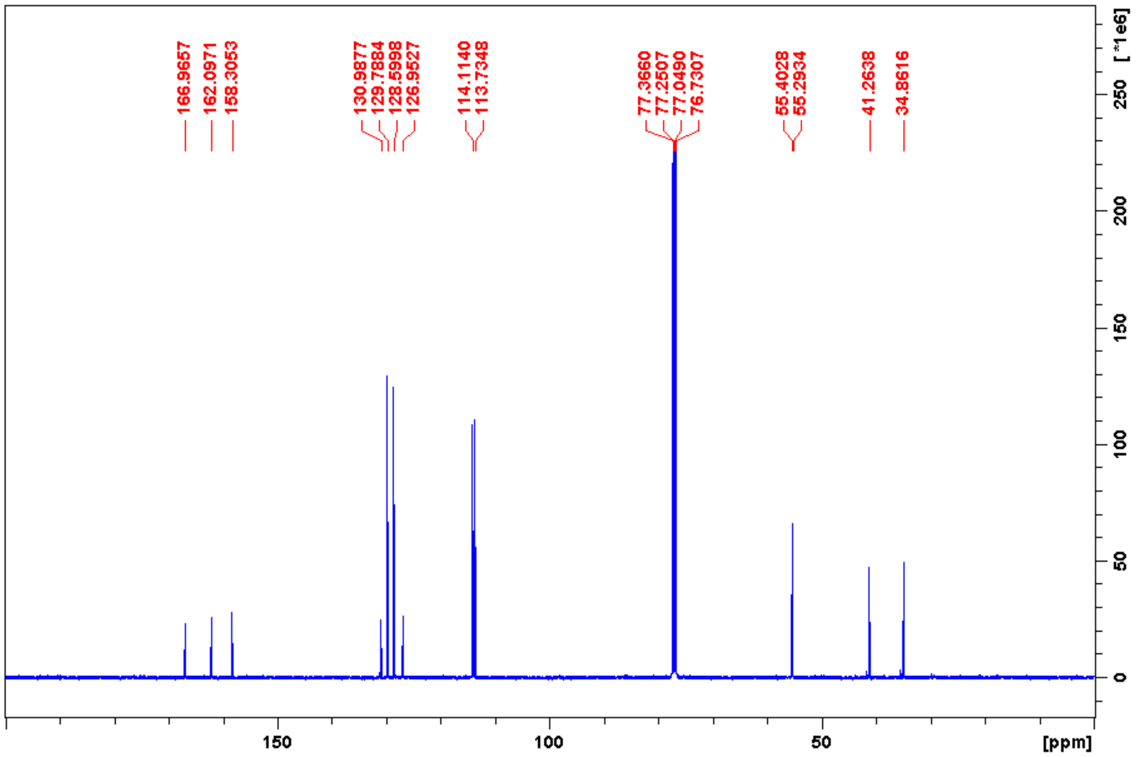

*N-(3,4-dimethoxyphenethyl)-4-methoxybenzamide, (**11**).*

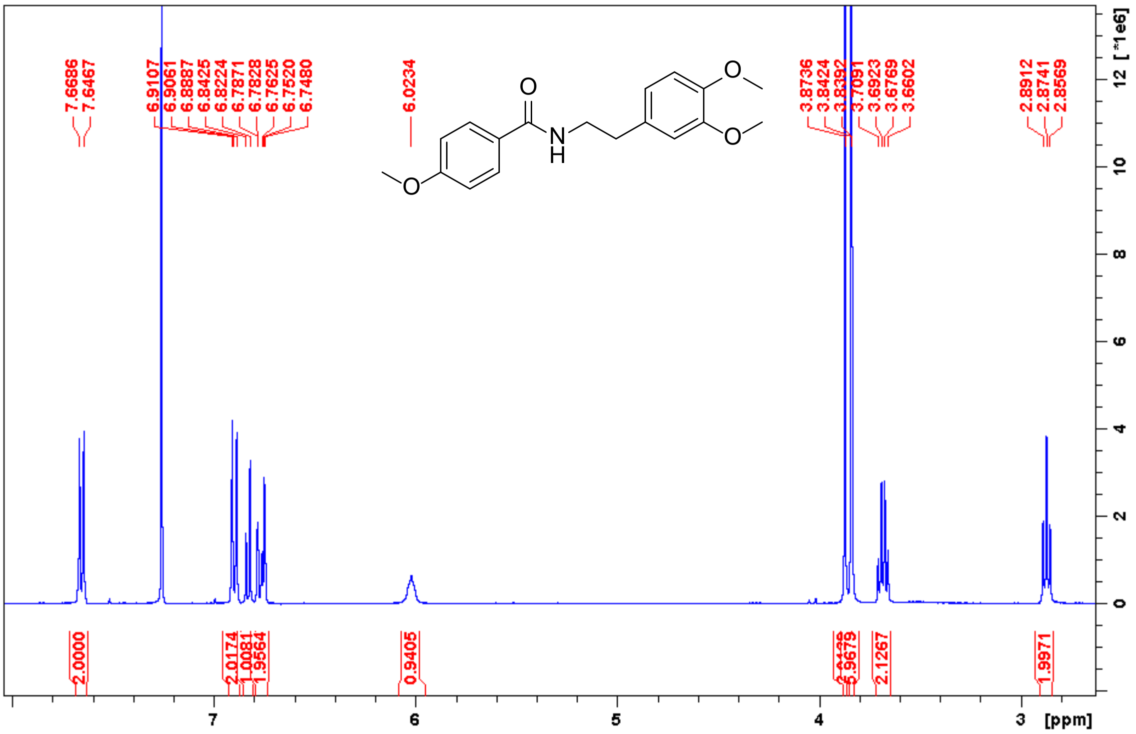

*N-cyclohexyl-4-methoxybenzamide, (**12**).*

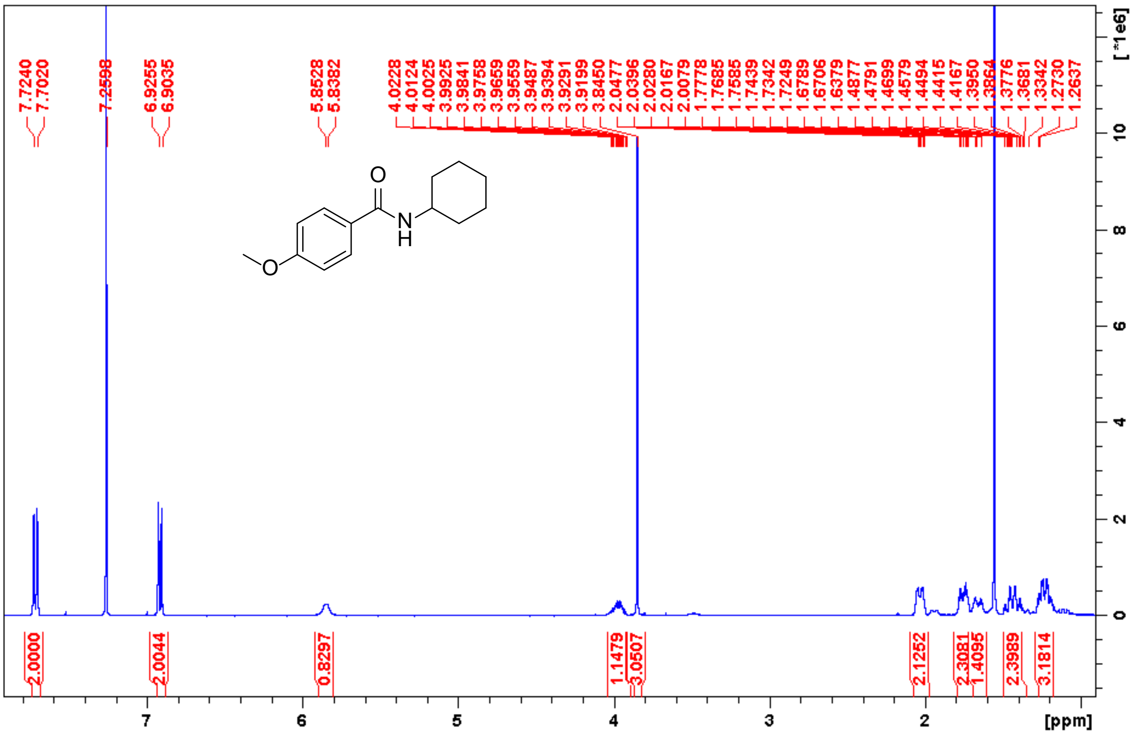

*4-Methoxy-N-(pyridin-3-ylmethyl)benzamide, (**13**).*

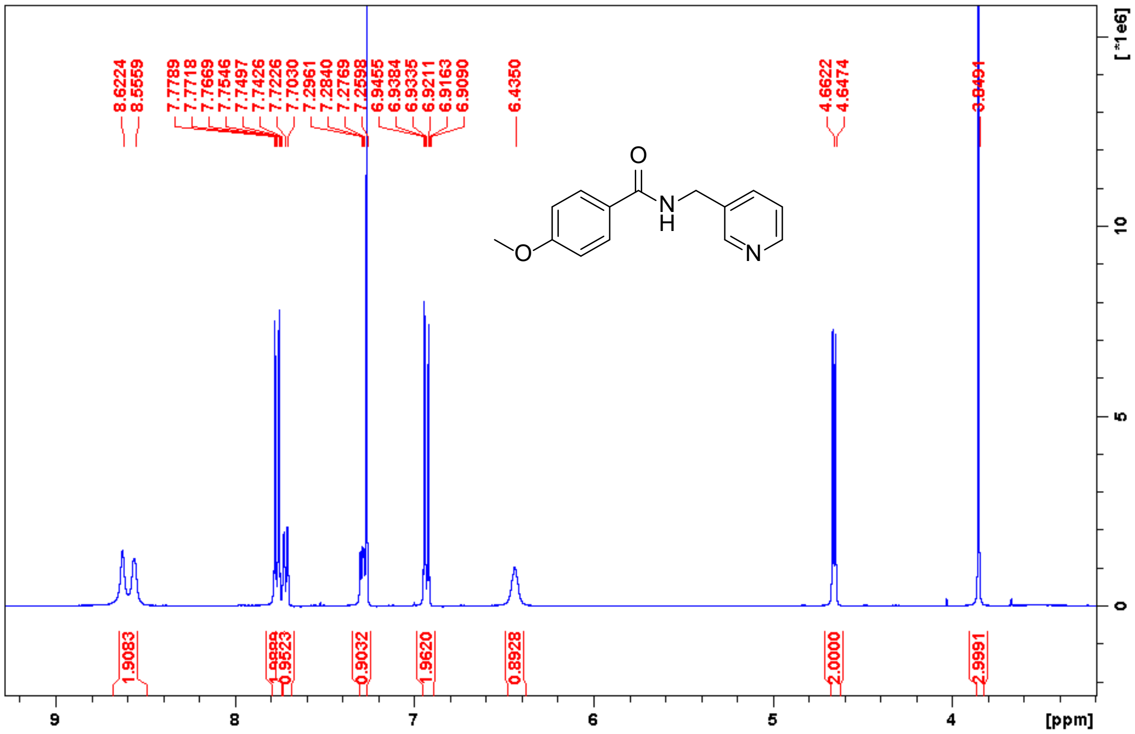

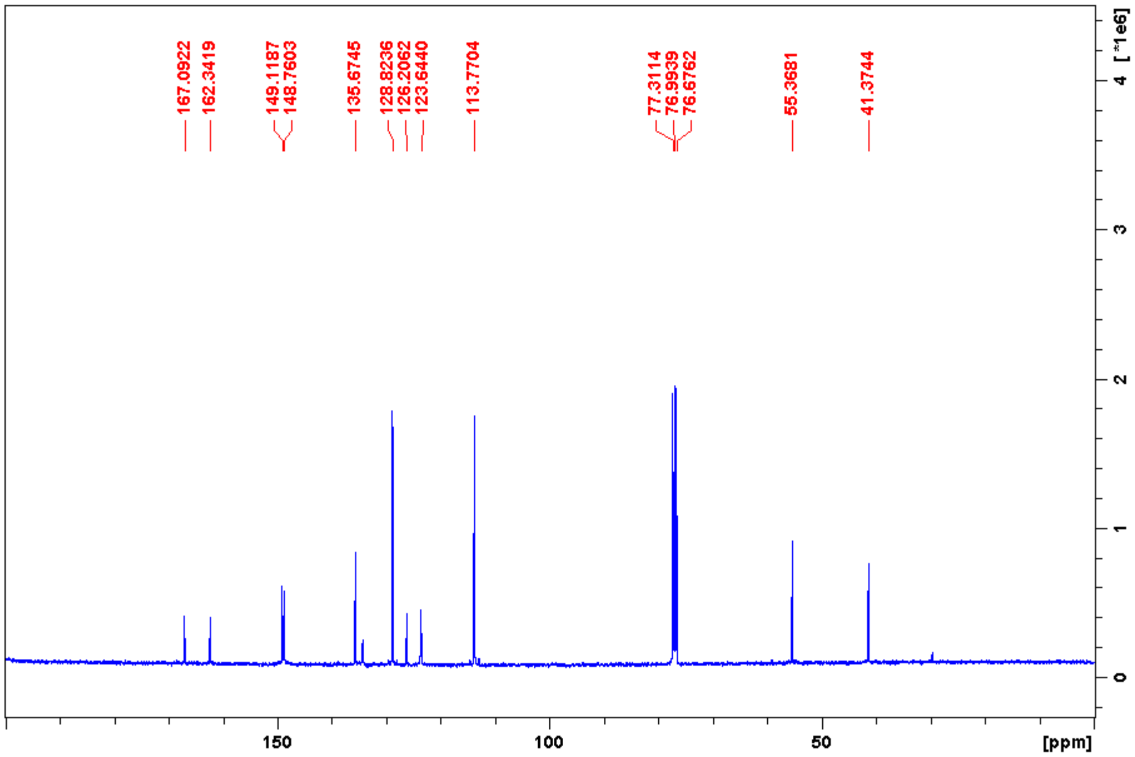

*4-methoxy-N-(pyridin-2-ylmethyl)benzamide, (**14**).*

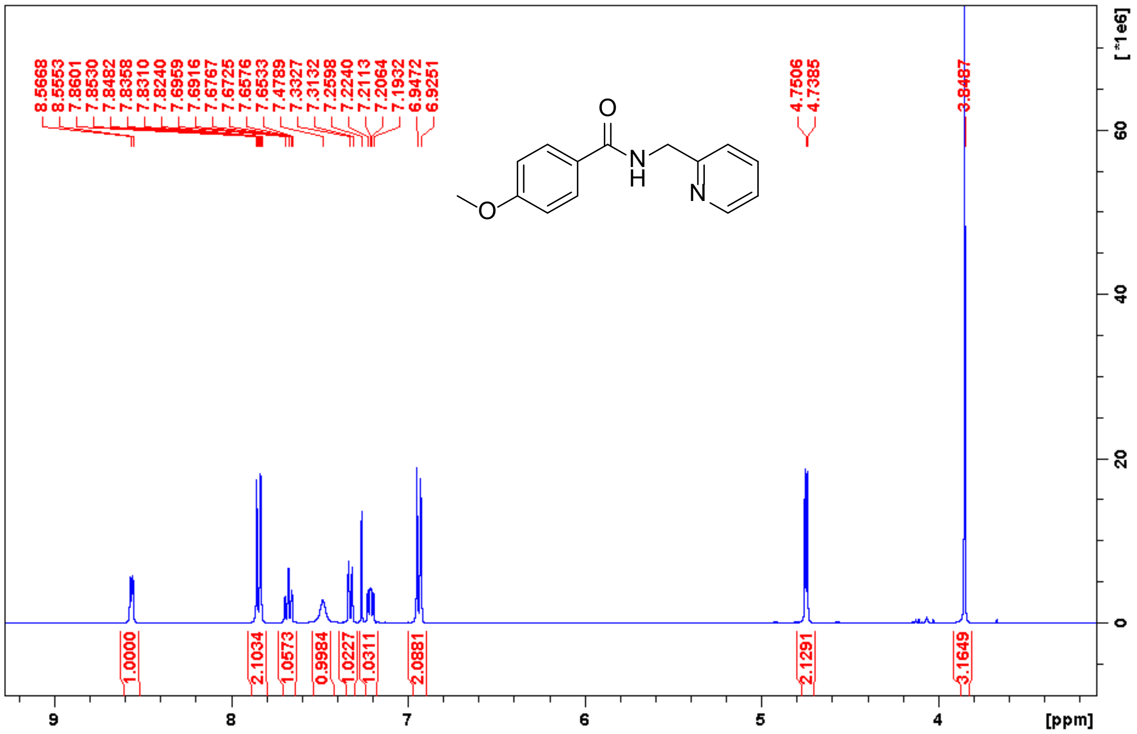

*4-Methoxy-N-(3-morpholinopropyl)benzamide, (**15**).*

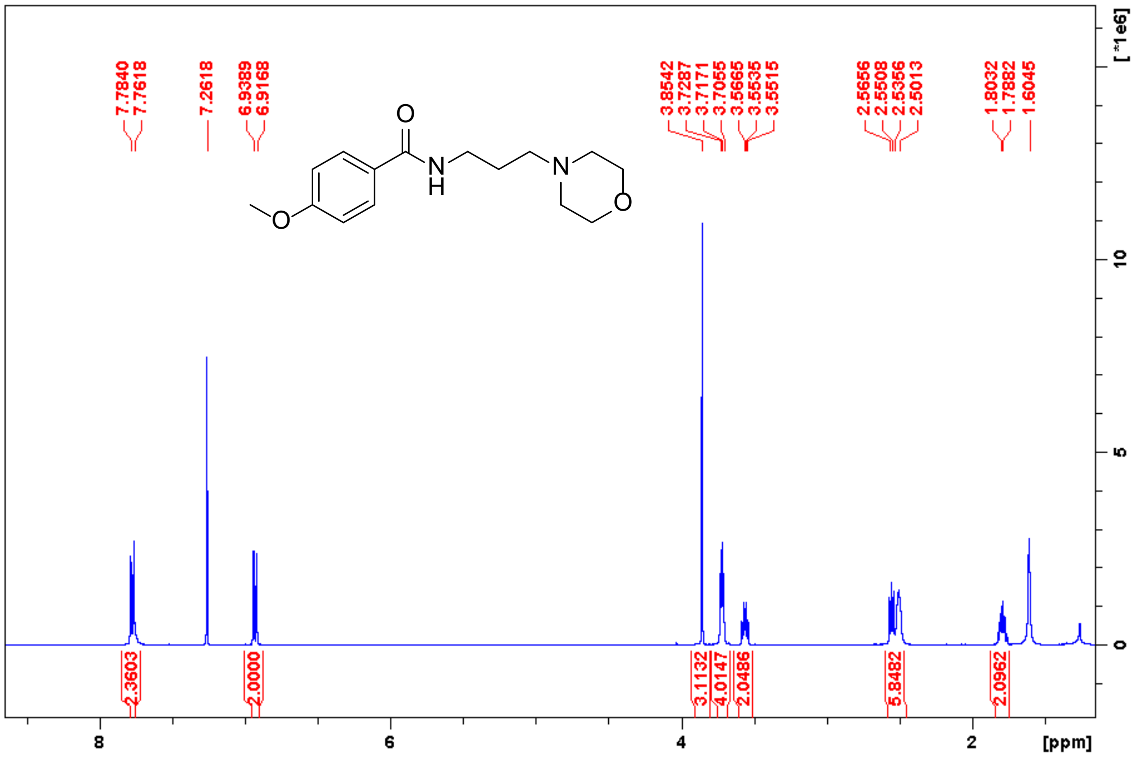

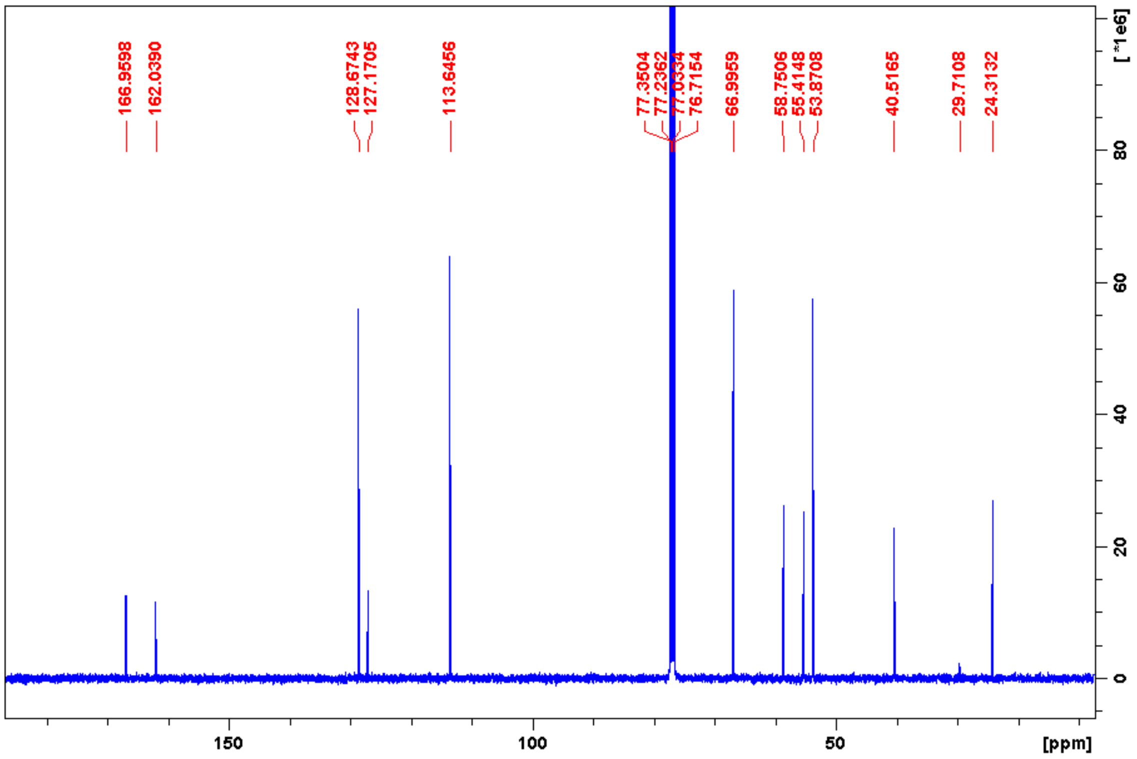

*N-(2-(1H-indol-3-yl)ethyl)-4-methoxybenzamide, (**16**).*

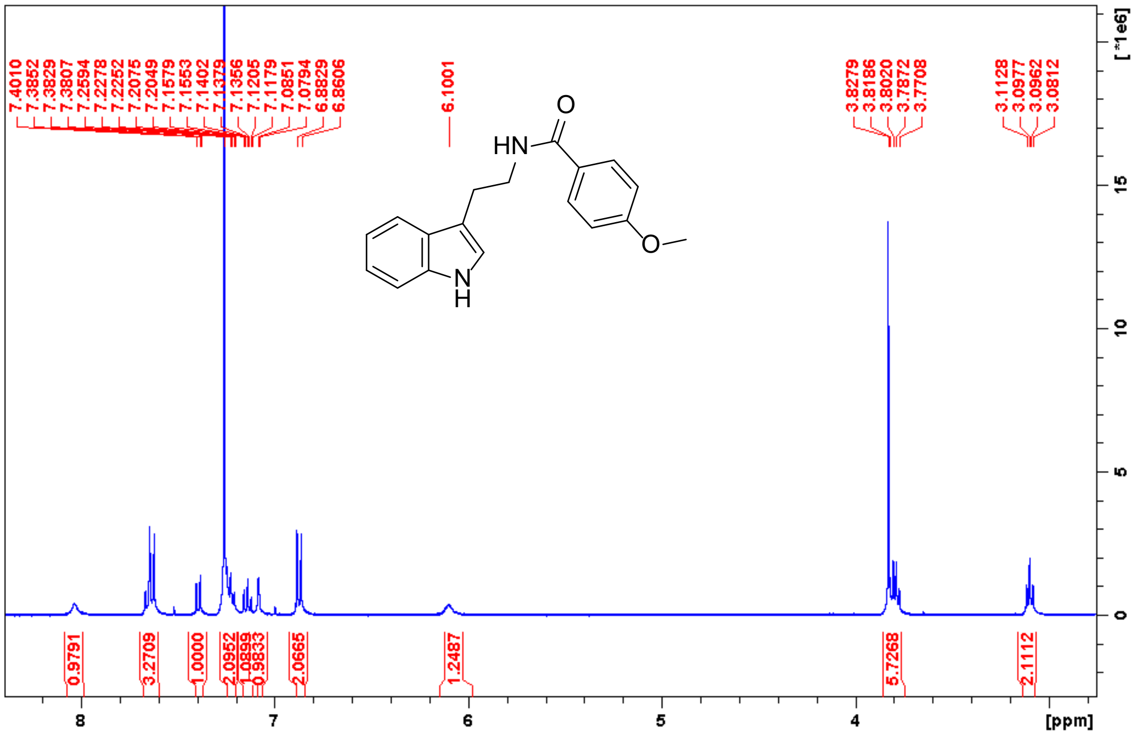

N-(4-isopropylphenyl)-4-methoxybenzamide, (**17**).

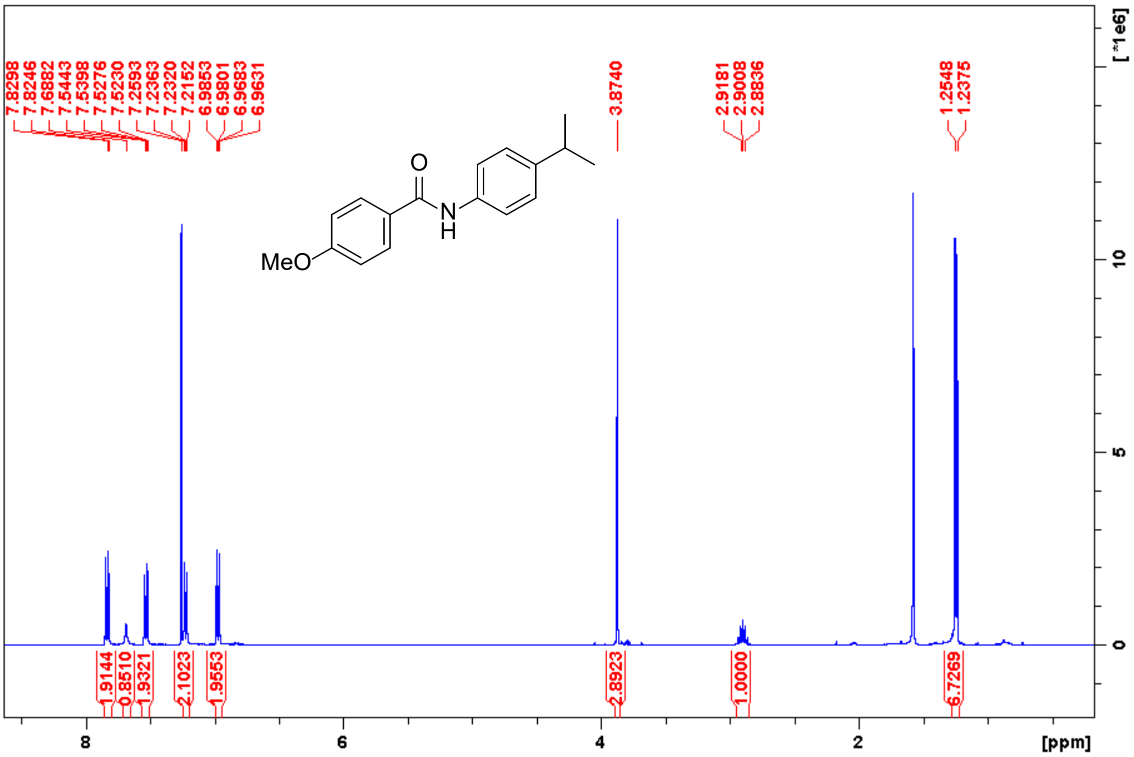

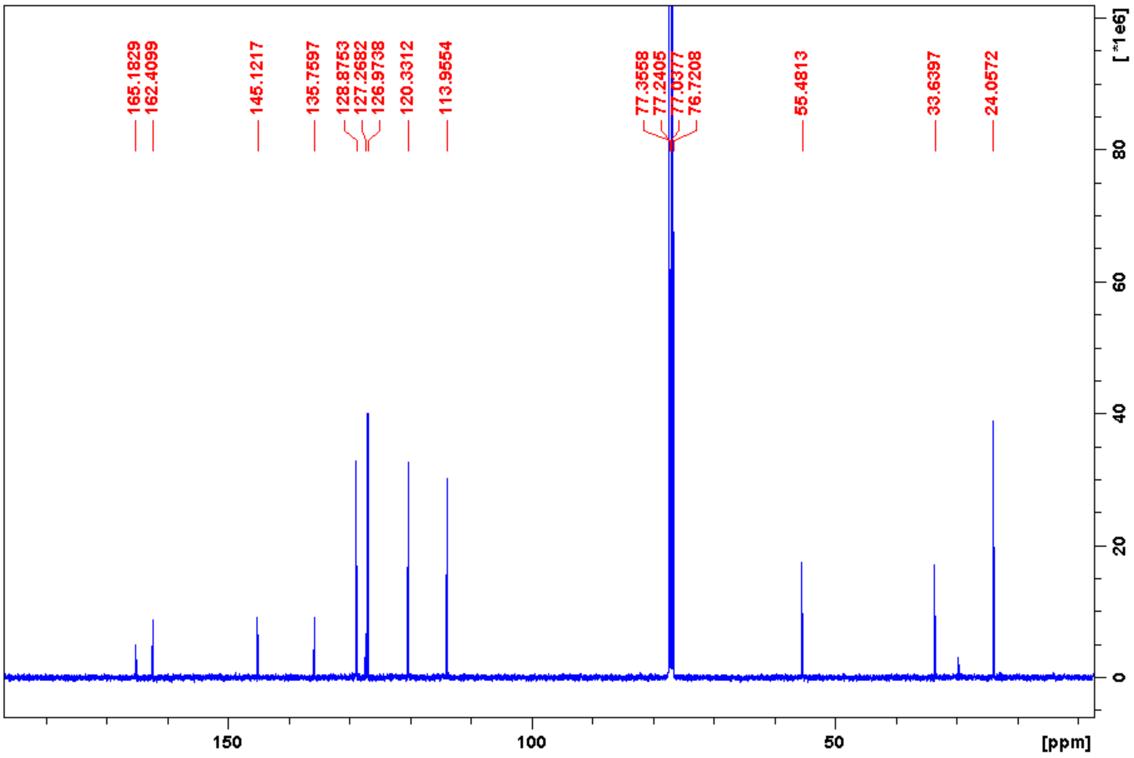

*N-(4-methoxyphenyl)4-methoxybenzamide, (**18**).*

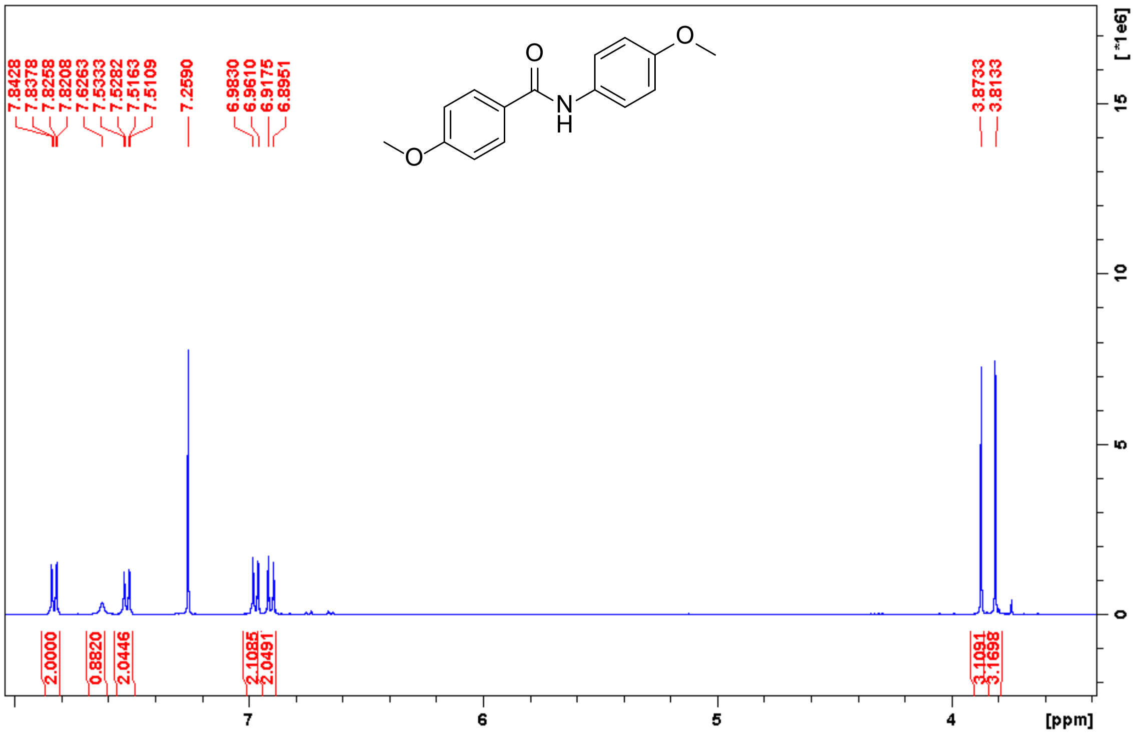

*N-(4-fluorophenyl)4-methoxybenzamide, (**19**).*

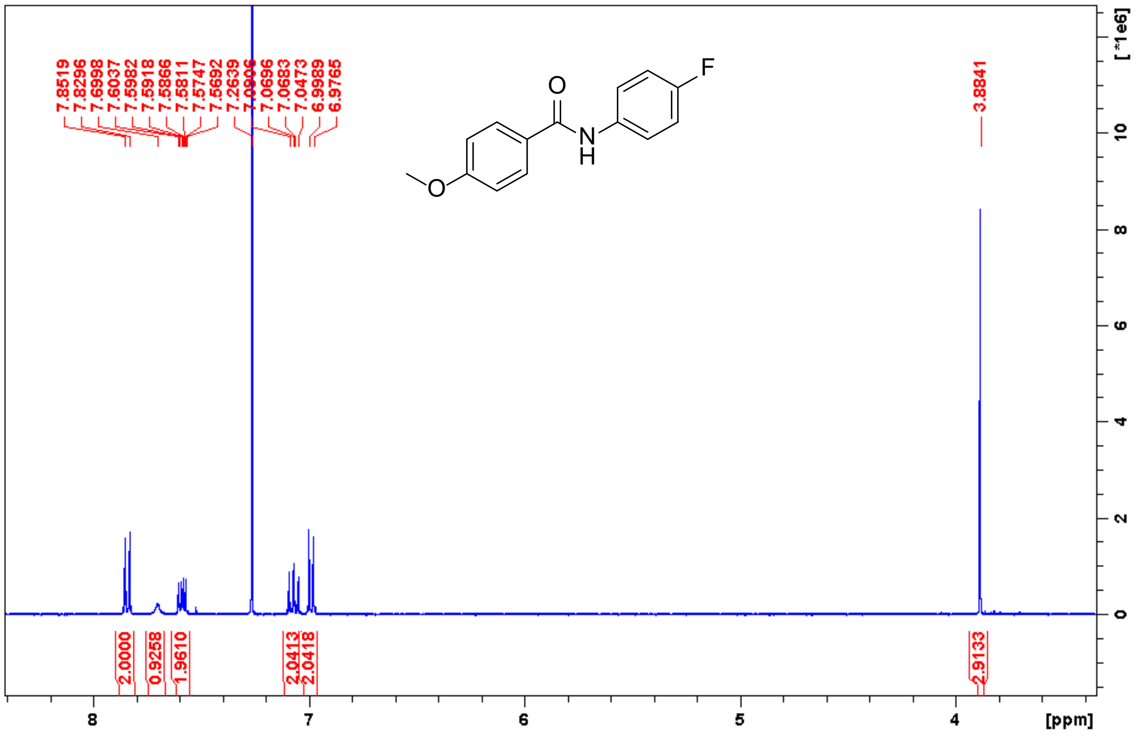

*N-(4-chlorobenzyl)benzamide, (**20**).*

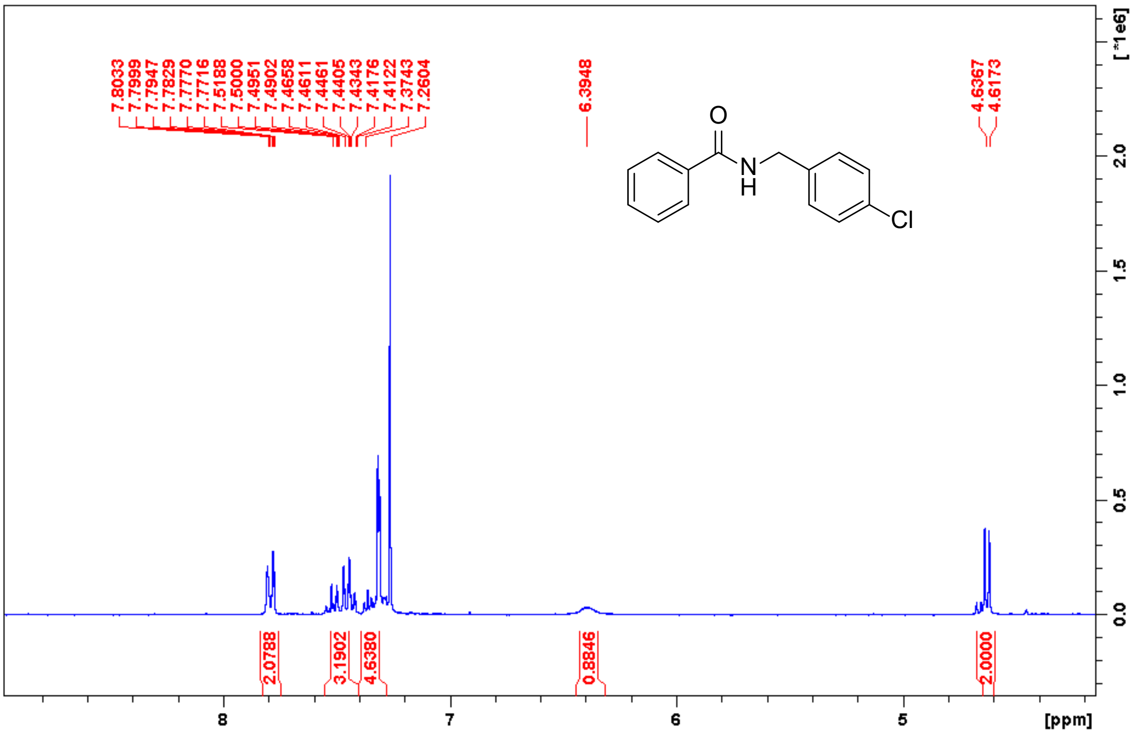

*4-bromo-N-(4-chlorobenzyl)benzamide, (**21**).*

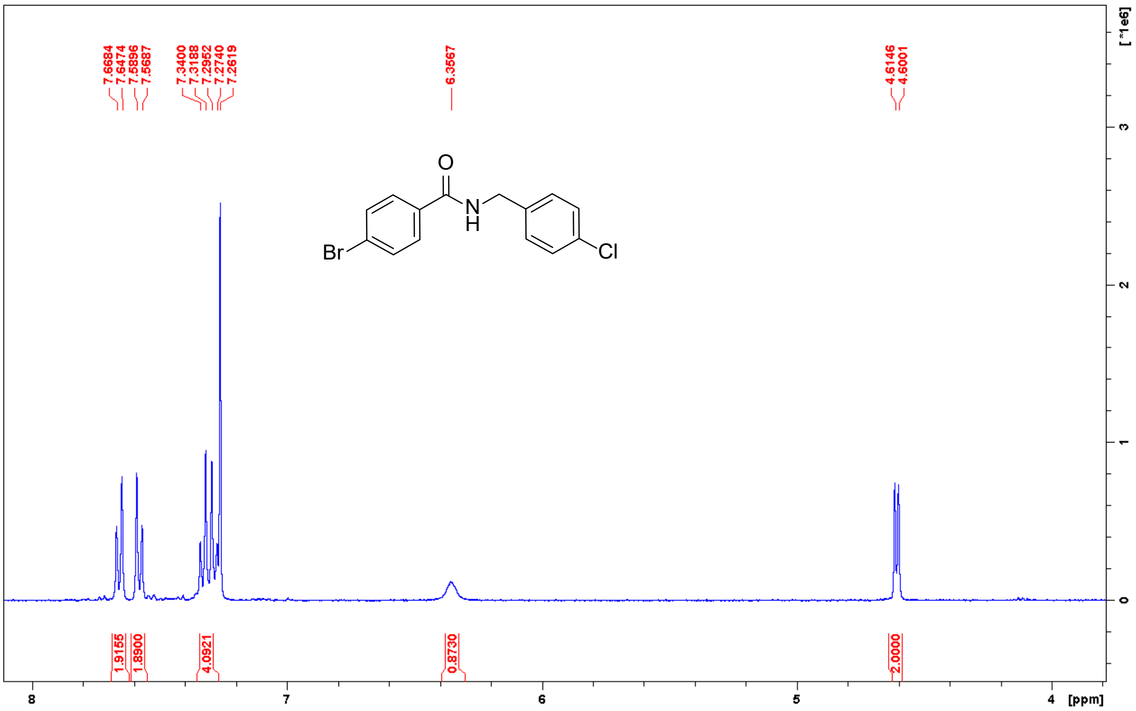

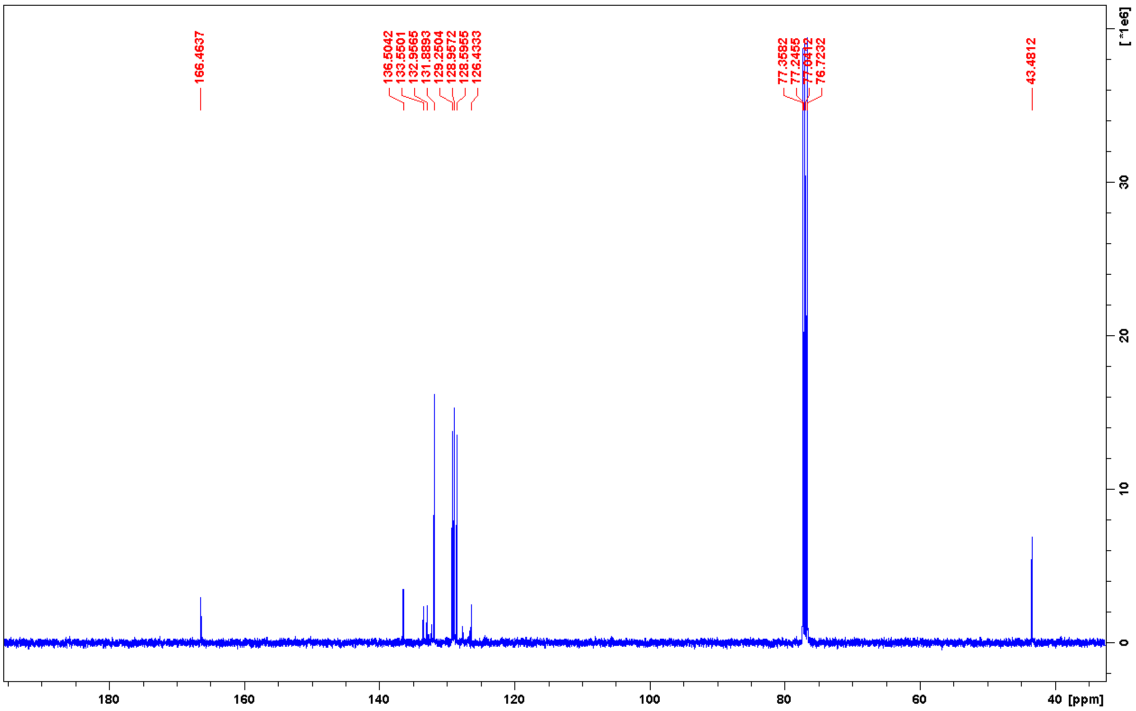

*N-(4-chlorobenzyl)-2-phenylacetamide, (**22**).*

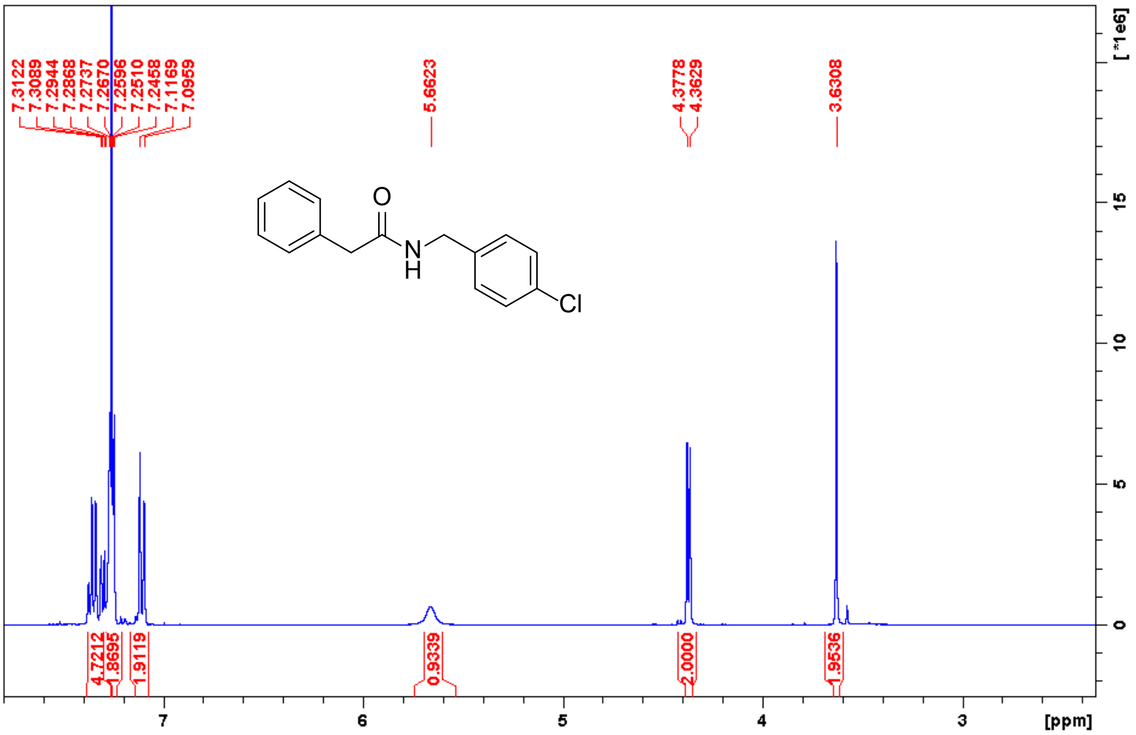

*N-(4-chlorophenethyl)-2-phenylacetamide, (**23**).*

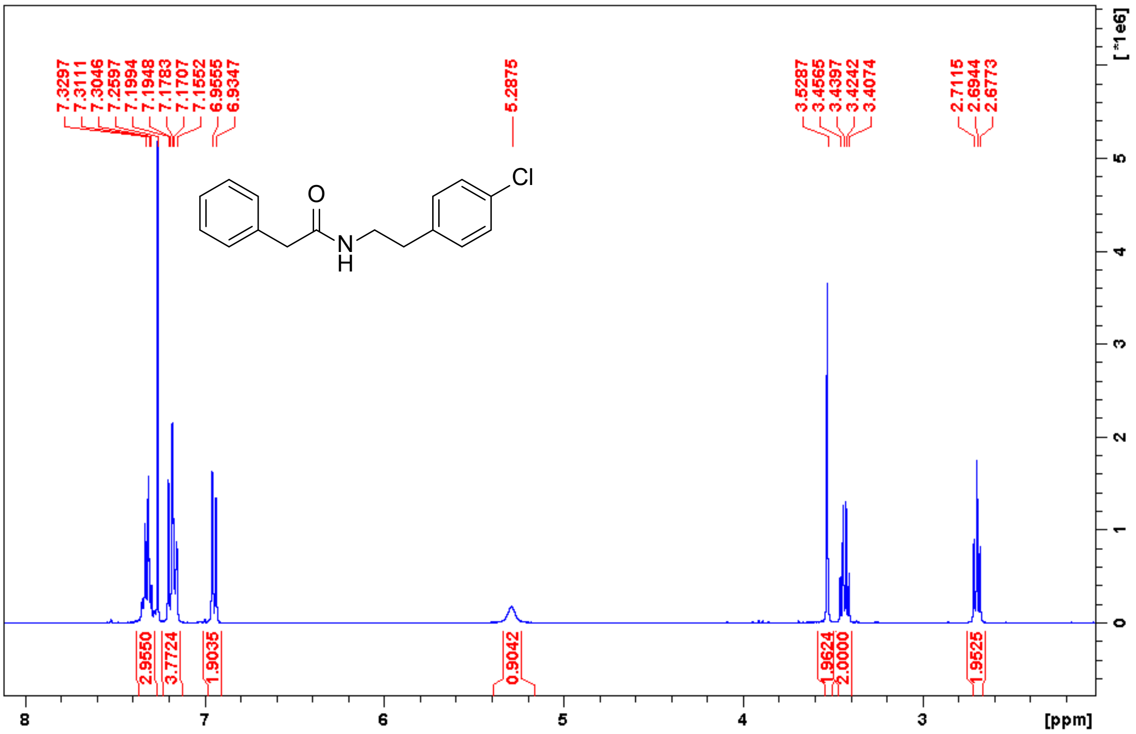

*N-(4-chlorophenethyl)pentanamide, (**24**).*

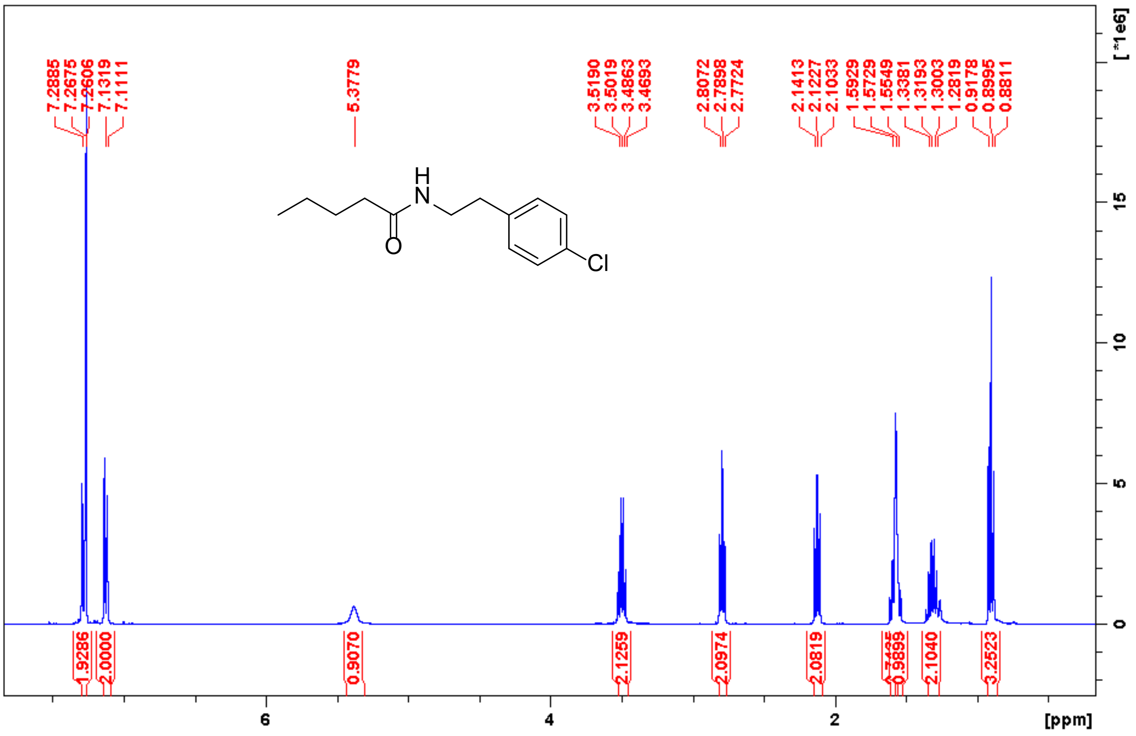

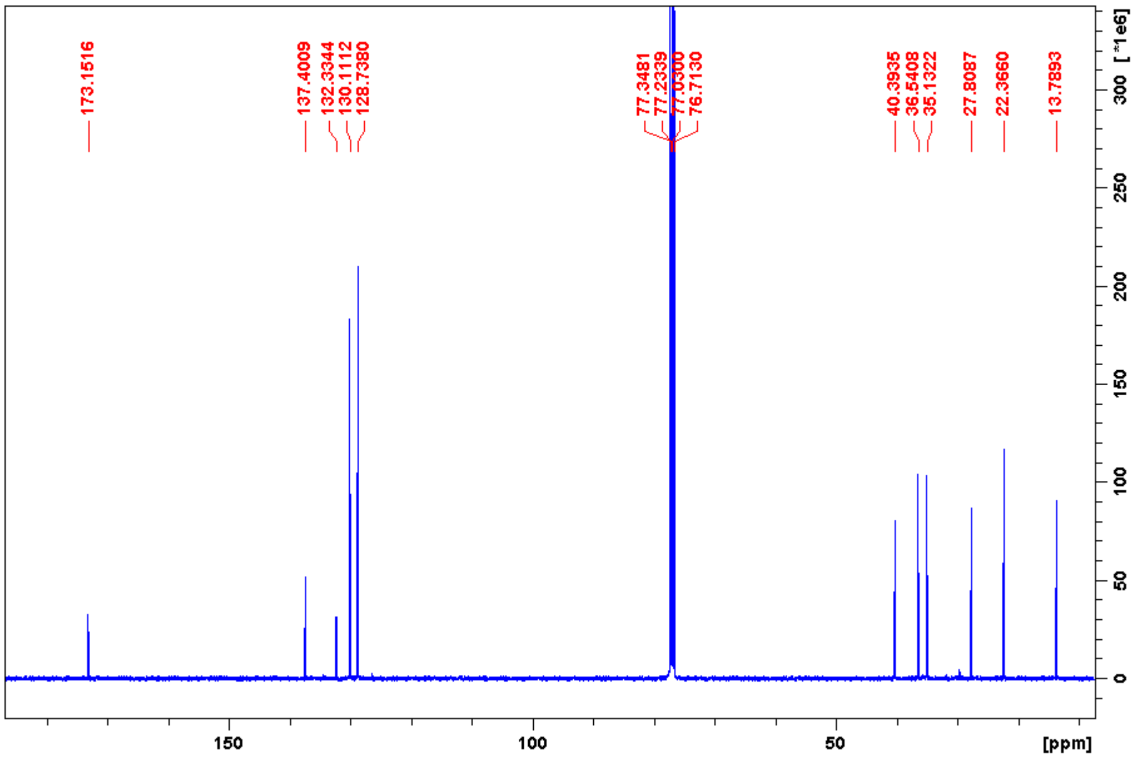

*N-(4-chlorobenzyl)cyclohexanecarboxamide, (**25**).*

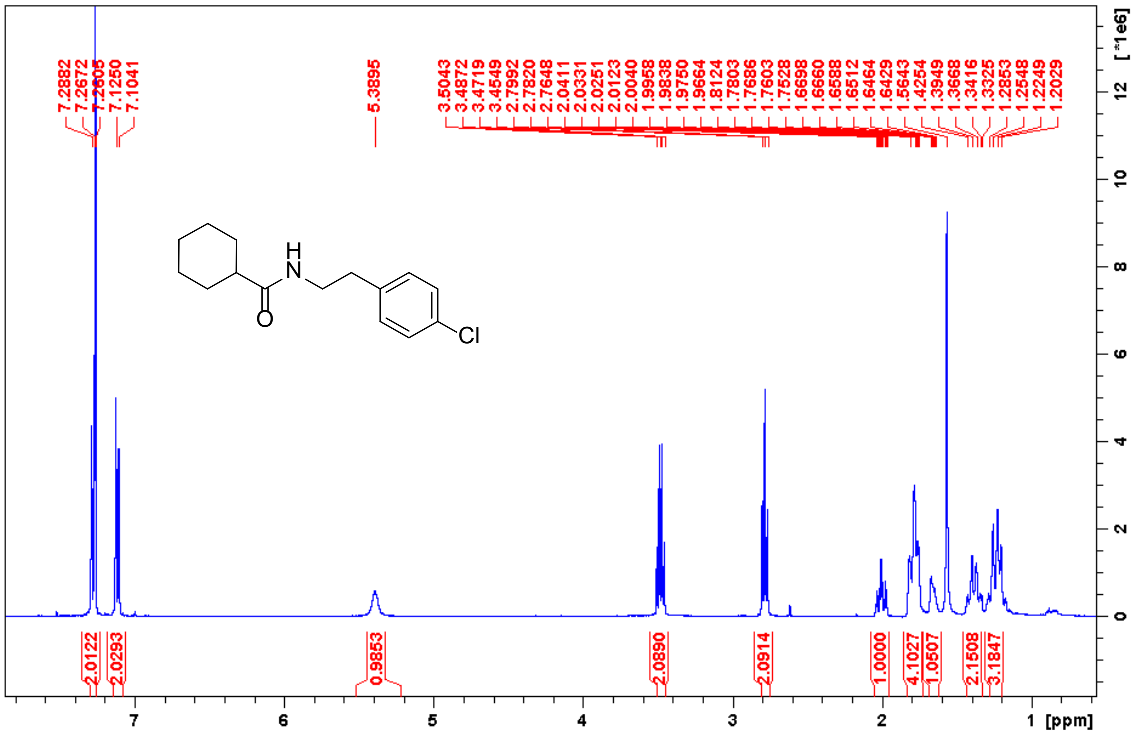

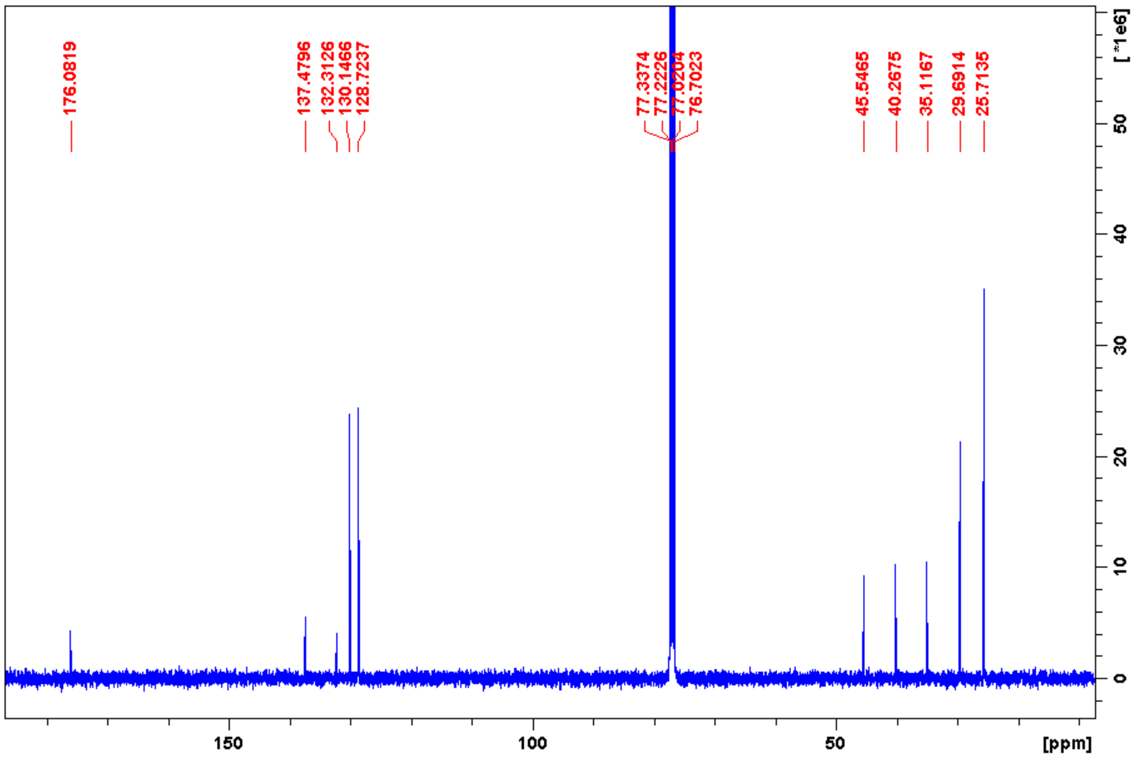

*N-(4-chlorobenzyl)cyclohexanecarboxamide, (**26**).*

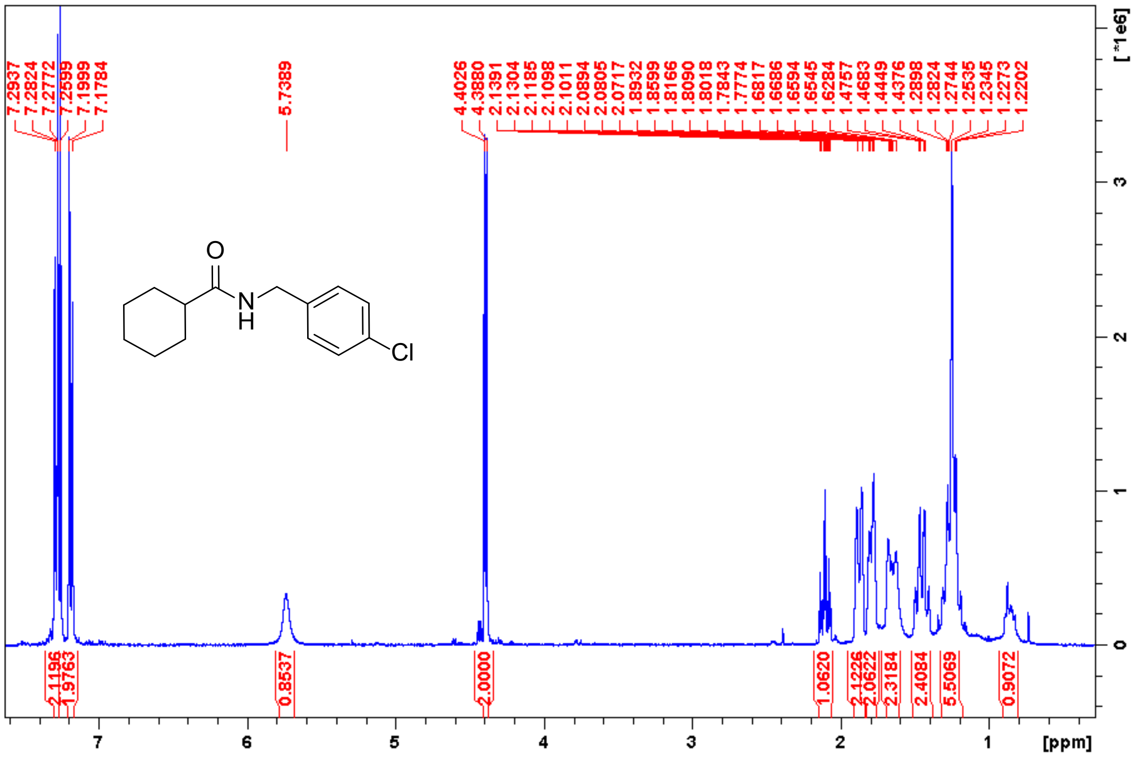

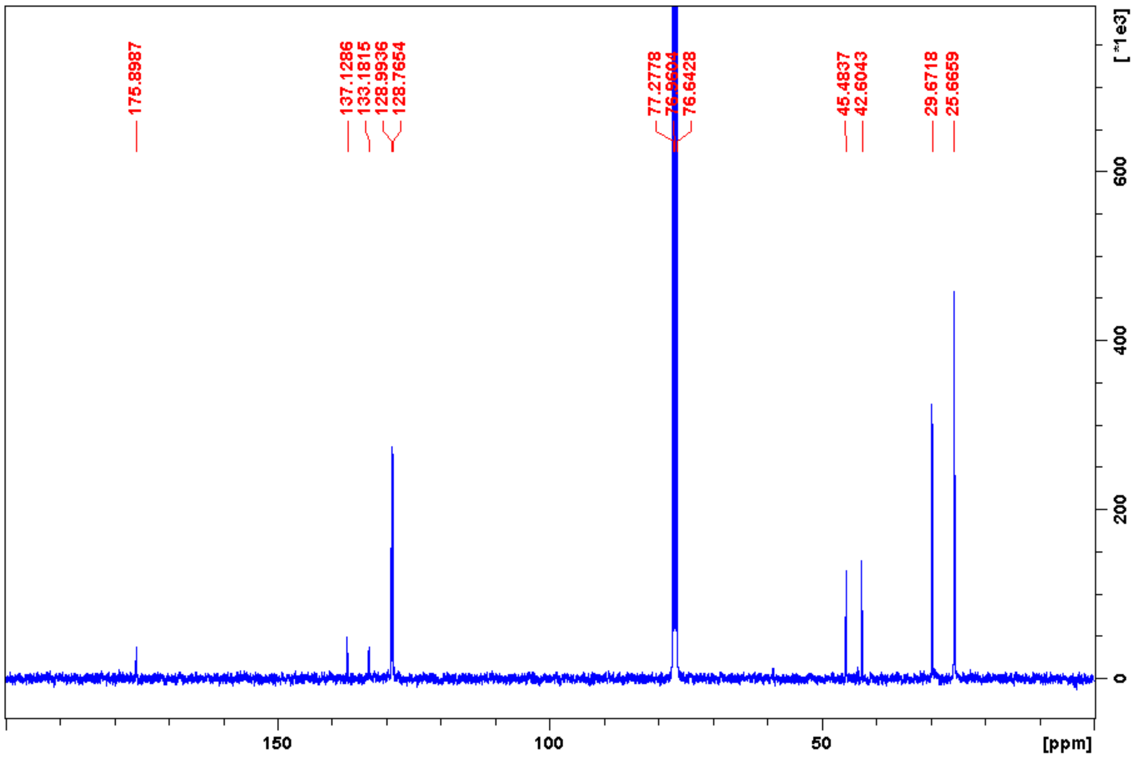

*4-chloro-N-(2-morpholinoethyl)benzamide (**moclobemide**).*

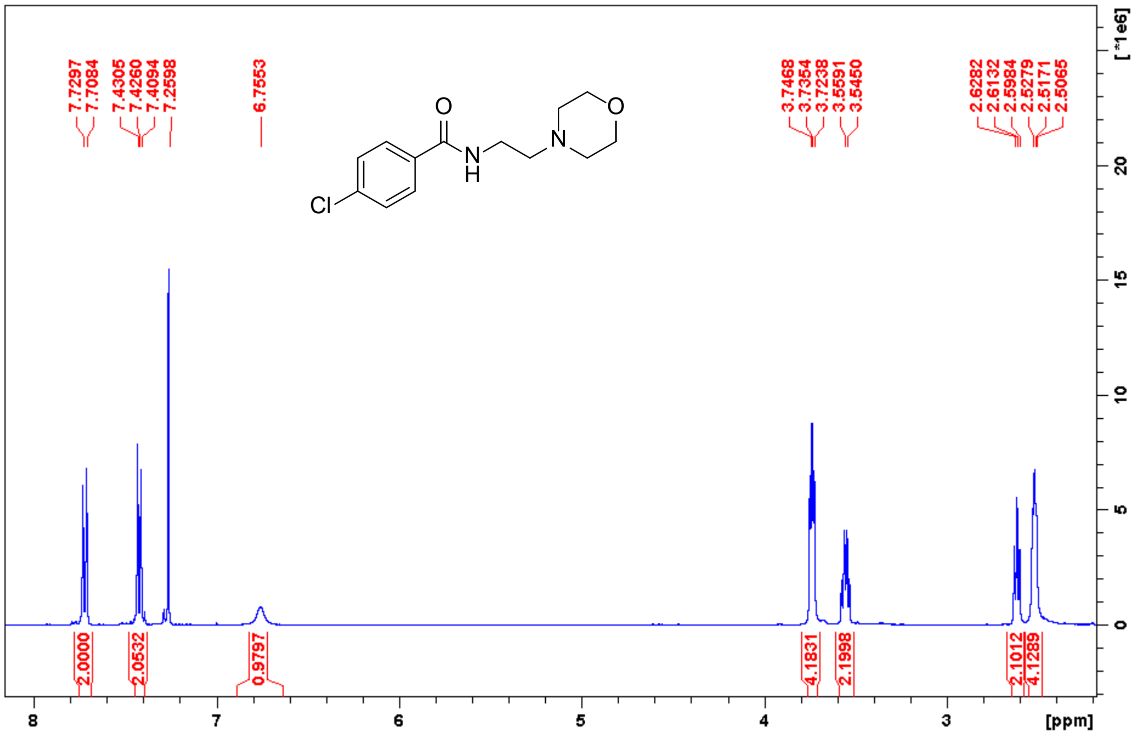

*N-(4-((2-(dimethylamino)ethyl)amino)benzyl)-3,4-dimethoxybenzamide (**itopride**).*

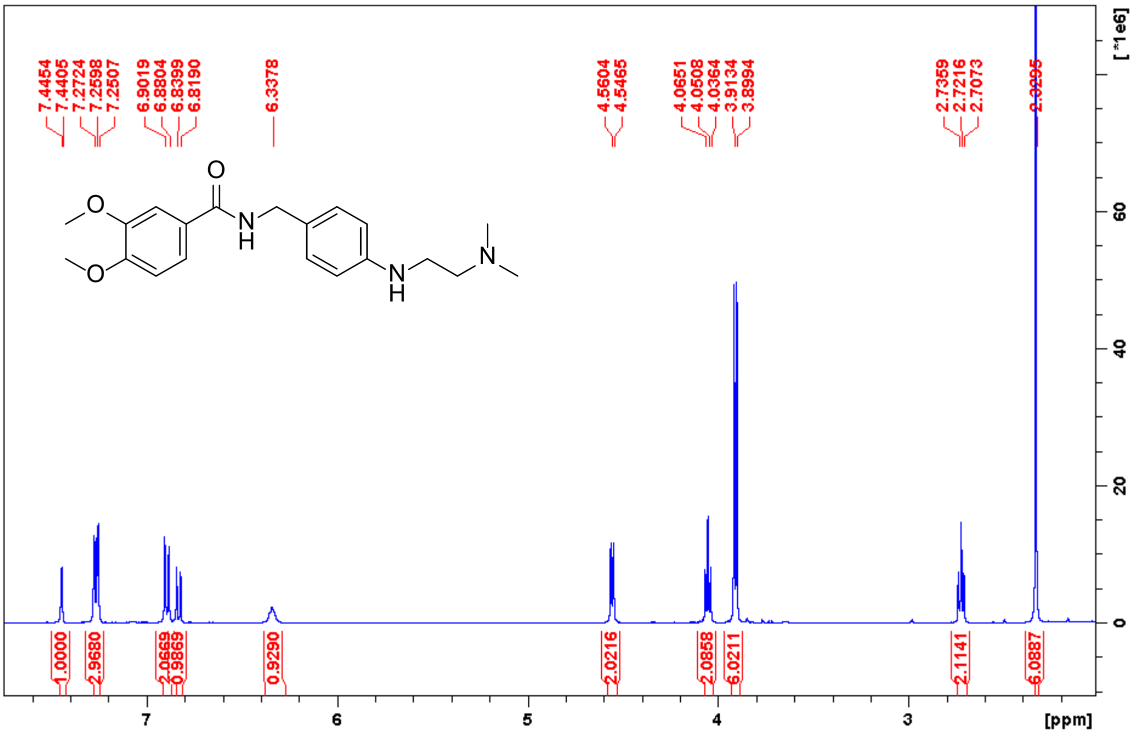

Supplement: RA-011-D1RA04736A-s001 [file RA-011-D1RA04736A-s001.pdf]
